# Application of a quantum crystallographic protocol to YLID, the world's most common crystal structure

**DOI:** 10.1038/s41598-025-95269-3

**Published:** 2025-04-29

**Authors:** Yaser Balmohammadi, Lorraine A. Malaspina, Yuiga Nakamura, Georgia Cametti, Michał Andrzejewski, Milosz Siczek, Simon Grabowsky

**Affiliations:** 1 Department of Chemistry, Biochemistry and Pharmaceutical Sciences, University of Bern, Freiestrasse 3, 3012 Bern, Switzerland; 2 Japan Synchrotron Radiation Research Institute (JASRI), Sayo-Cho, Hyogo, 679-5198 Japan; 3 Institute of Geological Sciences, University of Bern, Baltzerstrasse 3, 3012 Bern, Switzerland; 4 Paul Scherrer Institute, Forschungsstrasse 111, 5232 Villigen, Switzerland; 5 Faculty of Chemistry, University of Wrocław, F. Joliot-Curie 14, 50383 Wrocław, Poland

**Keywords:** X-ray diffraction, Materials chemistry

## Abstract

2-Dimethylsulfuranylidene-l,3-indanedione (YLID) is the most common crystal structure in the world not because of its chemical or physical properties, but because of its use as a test crystal for commercial diffractometers by nearly all vendors for more than 50 years. We will use it as an example here to showcase how the application of modern quantum-crystallographic refinement techniques and new experiments can unravel a so-far hidden story, which puts the emphasis back on the interesting chemical and physical properties of this crystal structure. We present a new chiral form of orthorhombic YLID (the left-handed *LS* form) and describe the complicated relationship between helical crystal packing and molecular planar chirality. We investigate polymorphs of YLID with twisted and planar molecular configuration as a function of temperature (100 to 292 K) and external pressure (0 to 4 GPa). However, finally only chemical pressure, namely the insertion of water into the crystal structure, can transform the twisted into the planar structure. A combination of quantum crystallography and repeated measurements of the orthorhombic test crystal gives access to an estimate of reproducibility and reliability of refining both anomalous dispersion and Flack parameters. It appears that the chemical environment of covalently bonded atoms has an influence on the anomalous dispersion parameters.

## Introduction

**Fig. 1:**
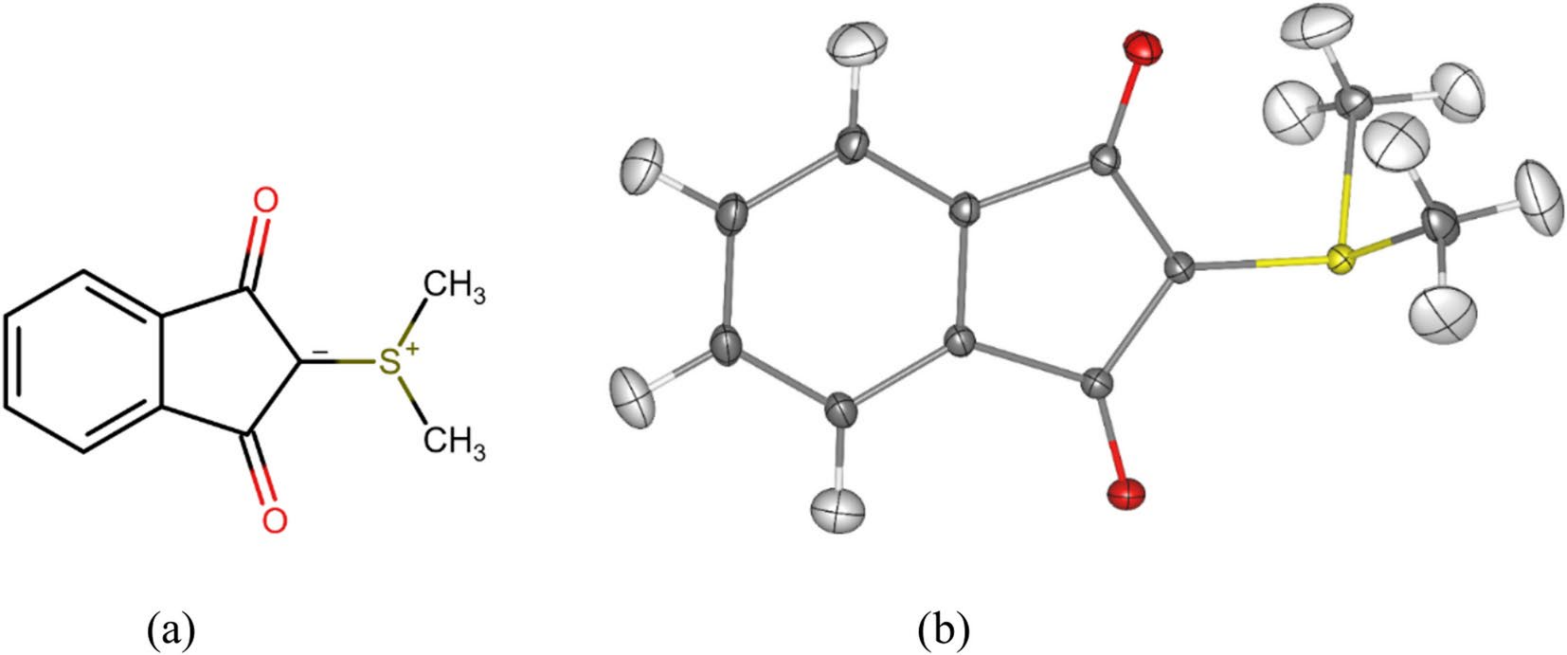
(**a**) The molecular structure of 2-dimethylsulfuranylidene-l,3-indanedione (YLID) in its most relevant Lewis resonance form. (**b**) Molecular structure of YLID from Hirshfeld Atom Refinement (HAR) of the orthorhombic crystal structure measured with Ag wavelength at 100 K (dataset 1, see Table S1). All anisotropic displacement parameters (ADPs) represented at a 50% probability level. Atomic labelling scheme in Figure S1.

Single-crystal X-ray diffraction is the foremost technique for atomically resolved three-dimensional structure determination. In-house X-ray diffractometers are nowadays available everywhere around the world offered as encased, radiation-proof solutions by various companies. Curiously, the test crystal to adjust and calibrate diffractometers is always the same: 2-dimethylsulfuranylidene-l,3-indanedione (YLID)^1^ (Fig. 1) in its orthorhombic polymorph. It has been provided to customers since 1969 by Bruker and Rigaku as well as the earlier Syntex, Nicolet, Enraf–Nonius, Oxford Diffraction, Kuma, and Siemens Analytical Instruments^2,3^. Although it was arbitrarily chosen, YLID has a few advantages as it is a small organic molecule stable under a range of conditions and it can be ground to spheres easily. Because of its use as standard, tens of thousands of YLID crystal structures must have been determined under various conditions and setups everywhere around the world over the past half a century. It is undoubtedly the world’s most common crystal structure.

Despite its abundance, there are only five studies in the literature that describe or use its crystal structure^2,4–7^ three additional communications in the Cambridge Structural Database (CSD)^8–10^, and only eight sets of available experimental structure factor files of the orthorhombic polymorph. We have recently contributed to this collection with 23 new measurements under different conditions^11^, motivating the submission of all those future YLID test measurements to build up a data base of repeated measurements that can be used for statistical analysis and method development. However, during the course of that study it became clear that there are many more findings to be made and lessons to be learned even from this most abundant crystal structure. Maybe because it is such a common structure, its chemical, physical, and crystallographic properties and characteristics have been widely overlooked? We present i) new quantum-crystallographic methodology and ii) new experiments that help to unravel the hidden story.

- i) New quantum-crystallographic methodology
- Quantum crystallography (QCr) encompasses all such methods and techniques of crystallography that aim to study quantum phenomena, such as chemical bonding, by exceeding the Independent Atom Model (IAM)^12–15^. In the IAM, atoms are considered as non-interacting spherical electron-density distributions, whereas in QCr atoms are non-spherical entities that follow quantum–mechanical rules. Hence, improved geometries, especially for hydrogen atoms^16^, experimentally reconstructed electron densities^17^ and wavefunctions^18^ can be determined with QCr means that are directly related to chemical bonding and materials properties^19,20^. With ever advancing hardware and software, and consequently improving data quality of routine measurements, such methods are becoming accessible to non-experts. Therefore, we have recently introduced a quantum crystallographic protocol (QCP) for general use^11^.
- This QCP makes use of the quantum crystallographic method Hirshfeld Atom Refinement (HAR)^21,22^ in the NoSpherA2^23^ implementation. HAR is a regular least-squares refinement procedure of atomic positions and (anisotropic) displacement parameters, but the underlying atomic form factors are not derived from spherical atomic electron densities as it is regularly done in the IAM as used in, e.g., ShelxL^24^. Instead, the atomic form factors are Fourier transforms of non-spherical Hirshfeld atoms generated via stockholder partitioning^25,26^. Here, we have applied the QCP to all previously published orthorhombic datasets 1–23 (see Table S1) and the new determinations 24 and 25 (see point ii)). In addition, we have performed X-ray wavefunction refinement (XWR)^27^, which is a combination of HAR and subsequent X-ray constrained wavefunction (XCW) fitting^18,28,29^ upon fixed coordinates and ADPs (including higher order anharmonic terms) from HAR. Hence, XCW fitting gives access to experimentally reconstructed density matrices and allows us to study derived electronic properties of YLID.
- ii) New experiments
- Most owners of the spherical orthorhombic test YLID crystal are probably not aware of the chirality of their sample, as this information is not given when the crystals are delivered by the diffractometer companies. The molecule does not have a stereogenic centre, but the crystal packing is chiral due to a helical axis. The helicity of every individual YLID crystal must be determined unambiguously in order to refine the structure accurately. In the literature, so far only crystals of RS helicity (clockwise, right-handed screw/ helical axis) have been described. Here, we add the description of the LS crystal form (anti-clockwise, left-handed screw/ helical axis, see also Table S1), and we test how well the chirality can be established for YLID in the diffraction experiments across the different wavelengths with quantum-crystallographic means, as the underlying anomalous dispersion effect is wavelength dependent. We further refine the anomalous dispersion coefficients f′ and f″ during HAR for the first time for repeated measurements to check their reproducibility and relate the refined values to a chemical interpretation^30^.
- The YLID molecule is twisted out of plane in the chiral orthorhombic structure (P2_1_2_1_2_1_), whereas it is planar in its monoclinic polymorph (P2_1_/c)^2^. To further analyse the interplay between crystal packing and molecular geometry, we added a quantum-crystallographic refinement of the monoclinic polymorph. We further performed a high-pressure experiment with the orthorhombic polymorph at 7 different pressure points up to 4 GPa at the Swiss Light Source, Materials Science beamline, to study whether a phase transition upon planarization occurs by application of external pressure. In addition, we applied chemical pressure on the orthorhombic polymorph via solid-state reactions to form a new modification. This also shows that the orthorhombic test crystal can undergo some transition in mineral oil and is, hence, not as stable as thought before.

## Materials and methods

### Synthesis and crystal growth

2-Dimethylsulfuranylidene-1,3-indanedione (YLID) was synthesized using a one-step synthesis in accordance with a previously reported procedure by Lácová and Šišková,^31^ and us^11^. The compound was recrystallized from acetone to give yellow crystals of the orthorhombic polymorph, whereas crystals of the monoclinic polymorph were obtained by diffusion of ethyl ether into a solution of YLID in acetone.

### Measurements

23 measurements of the orthorhombic polymorph were conducted at varying temperature (100 K, 150 K, 295 K), different wavelengths (Ag, Mo, Cu, synchrotron SPring-8) and with different crystal shape (natural vs. spherical). Table S1 summarizes the conditions under which they were measured and the distribution of chiralities. A more detailed discussion and analysis of those 23 measurements is reported in reference 11. The monoclinic polymorph was measured at 100 K on a Rigaku Synergy-S instrument with a Cu microfocus source and HyPix-6000 hybrid pixel detector. The new modification (water co-crystal) was measured at 100 K on a Rigaku Synergy-R instrument with a rotating anode source and HyPix-Arc-100 hybrid pixel detector. A single crystal of the orthorhombic polymorph was loaded into a diamond-anvil cell^32^ and Daphne oil 7474 was used as a pressure transmitting medium (PTM) with a hydrostatic limit of 3.7 GPa^33^. Xray diffraction data for seven different pressure points (0.00, 0.12, 0.59, 2.12, 2.82, 3.23, 3.89 GPa) were collected using synchrotron radiation at the Materials Science Beamline of the Swiss Light Source (λ = 0.49225Å and Pilatus 6M detector)^34^. The last data point is slightly above the hydrostatic limit of the PTM, but diffraction was still very good. We attempted to collect an additional data point at approx. 5 GPa, but here diffraction was poor, indicating disintegration of the crystal under non-hydrostatic conditions, and no unit-cell parameters were determined. A summary of all measurements and refinements is given in Table 1, more details in Tables S2 and S3.

**Table 1:** Summary of X-ray data collection conditions and refinement results; HAR for **1**–**25**, and IAM for high pressure measurements **26**.

	Resolution (Å)/CCDC-no	Wavelength*	Temperature	Crystal/ Crystallographic Chirality	R-value	Residual density e/Å^3^ (max/min)
**1–23**	0.42–0.81/see Table S1	Cu, Mo, Ag, synchrotron SPring-8	292 K, 150 K, 100 K	Orthorhombic polymorph/*LS* and *RS*	See Table S1	See Table S1
**24**	0.575/2310173	Mo	100 K	New modification/not chiral	0.0261	0.455/-0.253^##^
**25**	0.784/2309634	Cu	100 K	Monoclinic polymorph/ not chiral	0.0151	0.170/-0.110
**26**	7 × high pressure/ 2321990–2321996	Synchrotron SLS	293K	Natural shape/*LS*	See Table S3	See Table S3

*Wavelength: Ag = 0.56087 Å; Mo = 0.71073 Å; Cu = 1.54184 Å; Synchrotron SPring8 = 0.2483 Å; Synchrotron SLS = 0.49225 Å. ^##^ This dataset is based on a twinned crystal. More information is given in Table S2.

All structures were solved with ShelxT^35^ and refined with ShelxL^24^ inside Olex2^36^ using the Independent Atom Model (IAM). For the high-pressure data sets, the IAM was the final model. Here, all non-hydrogen atoms were refined anisotropically and positions of hydrogen atoms were taken from the molecular geometry with U_iso_(H) = 1.2/1.5 × U_iso_(carrier atom) for methyl (AFIX 137) and aryl (AFIX 43) hydrogen atoms, respectively. Table S3 summarizes all crystallographic, measurement and refinement details of the high-pressure measurements. For all other data sets 1–25, quantum-crystallographic refinements were carried out according to the HAR and XWR strategy that was discussed in the Introduction. For 1–23, the full XWR protocol was used to model the experimental electron density. Details of the underlying refinement models were discussed in ref. 11 and are summarized in Table S1.

For this study, all data sets measured with Cu radiation (2,10,15,18–23) as well as the new alternative modification (24) and polymorph (25) were re-refined with HAR according to the quantum crystallographic protocol introduced in ref.^11^. For this purpose, unmerged data were used, and the software NoSpherA2,^23^ which combines the refinement engine olex2.refine^37^ and the quantum-chemical software Orca5.0^38^ within the graphical user interface Olex2^36^.^39^ The B3LYP functional^40,41^ and def2-TZVP basis set^42^ were chosen. All hydrogen atoms were refined freely and anisotropically. For datasets 2,10,15,18–23, the anomalous dispersion coefficients Δf′ and Δf″ were also refined in olex2.refine as part of the HAR model. The crystallographic information files (CIFs) along with the structure factor lists for all refinements based on HAR were submitted to the Cambridge Structural Database under deposition numbers CCDC-2309634 and -2310173. Moreover, the seven high-pressure datasets refined using the IAM were also deposited to the CCDC with deposition numbers 2321990 – 2321996. They can be obtained free of charge from https://www.ccdc.cam.ac.uk/structures.

### Theoretical Calculations

The experimental geometries of datasets 1 and 24 were chosen as starting points to perform DFT geometry optimizations for the respective structure in the isolated state at the B3LYP/def2-TZVP level of theory. In addition, two single-point calculations were performed for the molecule in the orthorhombic twisted and monoclinic planar conformations at the CCSD/def2-TZVP level of theory. The software Gaussian 09^43^ was used and the resulting wavefunction was analysed using the AIMALL software^44^.

## Results and discussion

### Chemical analysis of the ylide bond

Figures 1 (b) and 5 qualitatively show that with HAR all hydrogen atom positions and anisotropic displacement parameters (ADPs) can be refined freely to a meaningful result. The refined bond lengths involving hydrogen atoms are given in Table S4. The average C-H bond lengths are 1.087(2)/ 1.079(8)/ 1.083(14) Å for aromatic and 1.075(6)/ 1.074(9)/ 1.078(8) Å for methyl groups in the order orthorhombic polymorph/ new modification/ monoclinic polymorph. Reference values from neutron diffraction are C-H(aromatic) = 1.083(17) Å and C-H(methyl) = 1.077(26) Å^45^. This shows that the bond lengths do not vary across the different polymorphs and modifications per bond type, and that the HAR-refined results from X-ray diffraction agree with reference values from neutron diffraction across all temperatures, resolutions and wavelengths. With those accurate geometries in hand, the next step was a wavefunction and electron-density modelling according to the XWR procedure.

**Fig. 2:**
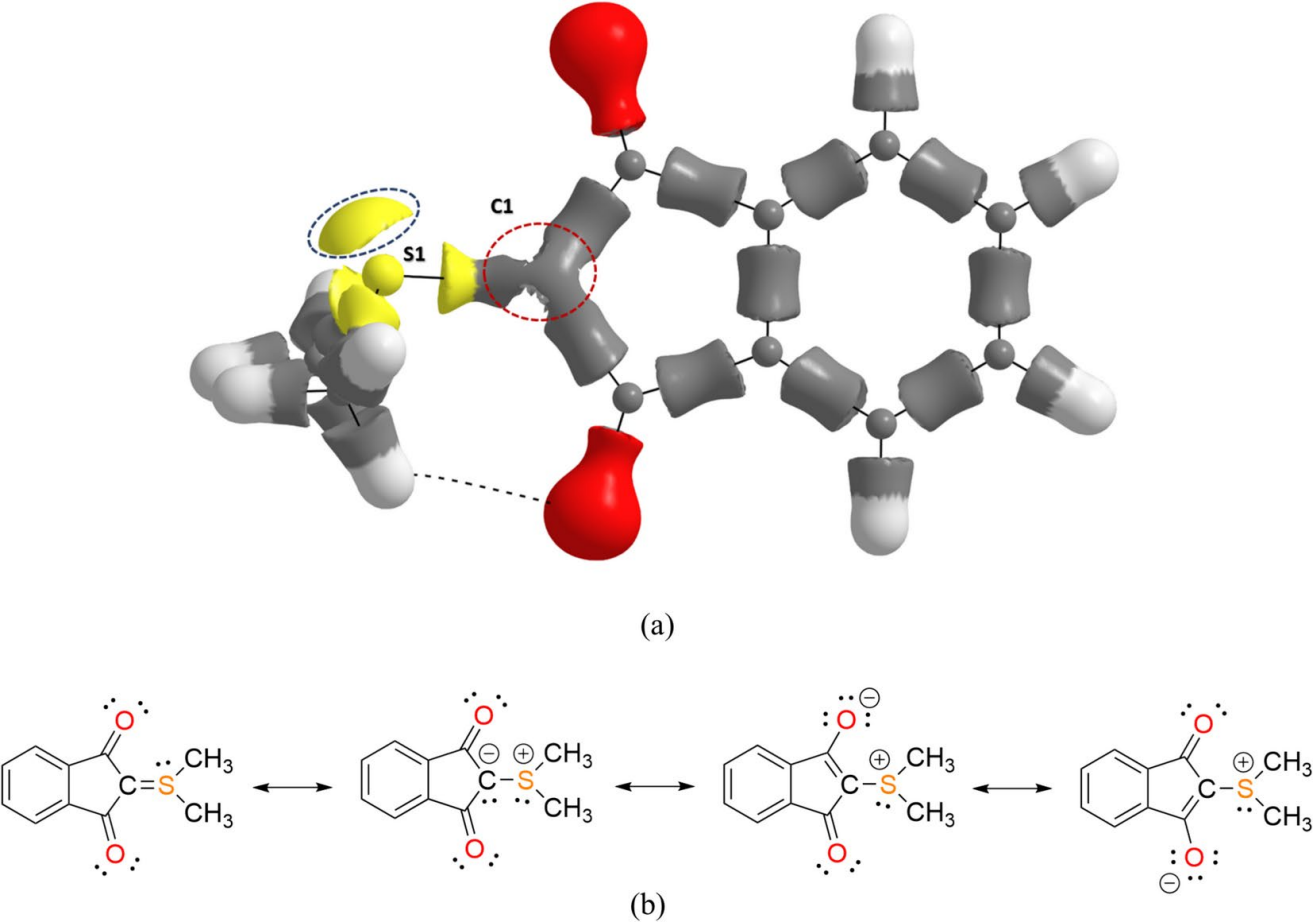
(**a**) Three-dimensional isosurface plot of the Laplacian of the electron density at an isovalue of -7.2 e/Å^5^. Blue-dashed and red-dashed circles represent the lone pair (charge concentration) of sulfur and carbon atoms, respectively. Density model: XWR of dataset 1. The colour scheme depicts the extension of the atomic basins; grey = carbon, white = hydrogen, red = oxygen, yellow = sulfur. (**b**) The four most contributing Lewis resonance structures.

Bader’s Quantum Theory of Atoms in Molecules (QTAIM)^46^ was applied to the experimental electron densities after XWR to obtain sets of descriptors related to chemical bonding and atomic properties. It was used for experimental studies of sulfur ylides before, but rarely^7,47,48^. A statistical analysis of the results for all data sets 1–23 was discussed in ref. 11. Here, we first concentrate on the chemical analysis by using only the results from data set 1. The S-C ylide bond, similar to the P–C ylide bond, can be expressed with two resonance forms, either the double-bonded S = C ylene form, which implies hypervalency at the S atom, or the charge-separated S^+^-C^-^ ylide form (Fig. 2). It is generally accepted that the charge-separated form prevails, which means that a lone pair must be found at the carbon atom in the electron-density distribution^49^. Figure 2 shows the three-dimensional distribution of the Laplacian of the electron density (also compare ref.^7^). Charge concentrations that depict lone-pair density are encircled. For the planar carbon atom, there is such density concentration symmetrically below and above the plane. In addition, the saddle-shaped region of the S-C bond is quite pronounced, e.g. in comparison to the C–C bonds, which implies ionic contributions to the bonding. However, the resonance system extends further to the carbonyl groups in this YLID molecule, adding two additional resonances (Fig. 2). This smears out electron density over a larger region, so that the carbon lone pair is not a stereoactive one.^7^ However, Bodensteiner et al. have shown very recently that a QTAIM analysis is not sufficient to determine the weights of the Lewis resonance structures reliably in sulfur ylides^50^. They highlight that the carbonyl-ylide structure is preferred over the enolate ones. Therefore, we will publish a more detailed complementary bonding analysis of the C-S, C-P, and C-As ylide bonds beyond the electron-density picture in a forthcoming article.

The bonding characteristics for the S-C_ylide_ bond (S1-C1) in terms of distances and QTAIM descriptors are given as an average over all 23 datasets modelled in XWR. One of the S-C_methyl_ bonds (S1-C11) serves as a reference for an S-C single bond without further ionic or π-bonding contributions. The S-C_ylide_ bond has a length of 1.708(2) Å, and is as such shorter than the S-C_methyl_ bond with 1.794(3) Å. However, both ionic and π-bonding effects would lead to a shortening. Therefore, we resort to electron-density descriptors. The averaged values of electron density and corresponding Laplacian at the S-C_ylide_ bond critical point (BCP) are 1.54(16) eÅ^-3^ and -12.1(35) eÅ^-5^, whereas they are 1.34(5) eÅ^-3^ and -9.7(14) eÅ^-5^ for S-C_methyl_. These values confirm two strong covalent bonds with slightly more electron density at S-C_ylide_, in agreement with the shorter distance, but no significant differences in the bond character represented by the Laplacian as the two values are the same in the standard deviation (SD). However, the ionic bonding aspect of the S-C_ylide_ bond is confirmed by the distribution of atomic charges. The sulfur atom is positively charged with 0.45(16) e in S-C_ylide_, whereas the carbon atom is negative with -0.24(7) e, so the charge separation is 0.70 e. For S-C_methyl_, the charge separation is smaller with 0.58 e, showing that ionic bonding contributions are more important in the ylide bond, however, the difference is again within a single SD when averaging across all 23 datasets.

### Reproducibility of refined anomalous dispersion coefficients

In every crystal structure refinement, for both non-centrosymmetric and centrosymmetric structures, anomalous dispersion coefficients *f*_*n*_*′* and *f*_*n*_*″* are used as correction terms to the atomic form factor *f*_*n,0*_ for each atom *n* in the structure according to    $f_{n} = f_{{n,0}} + f_{n}^{\prime } + if_{n} ^{{\prime \prime }}$. They describe the X-ray absorption and dispersion behaviour of the compound, and are strongly wavelength-dependent. In a recent publication, Bodensteiner et al. point out that X-ray absorption is specific to each atom in its local chemical environment, but anomalous dispersion coefficients *f′* and *f″* are calculated for isolated atoms without consideration of the spatial and electronic situation of an atom in a compound^30^. In Hirshfeld atom refinement (HAR), the chemical environment is included in the form factor *f*_*n,0*_ by the non-spherical Hirshfeld partitioning, so it is only consequential that for such accurate quantum-crystallographic refinements the environment should also be considered for *f*_*n*_*′* and *f*_*n*_*″*. Hence, refinement of *f*_*n*_*′* and *f*_*n*_*″* was implemented as parameters in the full-matrix least-squares procedure in olex2.refine and recommended to be used for NoSpherA2-type HARs^30^. However, due to the lack of available test datasets from repeated measurements of the same compound, the reproducibility and reliability of the refined *f*_*n*_*′* and *f*_*n*_*″* values have not been established yet.

Here, we go into that direction by refining these values during HAR in NoSpherA2 for the sulfur atom in all 9 Cu-K_α_ YLID datasets of the orthorhombic polymorph (Table 2). The Cu-K_α_ wavelength (1.5 Å) was chosen because the anomalous dispersion effect becomes stronger with longer wavelengths, except just at the absorption edge (K-edge for S = 5.0 Å). Such a reproducibility study would be more impactful for a heavier element closer to an absorption edge, but this depends on data availability. However, anomalous dispersion is already significant in charge-density quality datasets using quantum-crystallographic refinements of atoms of the second and third period^51^. The refined values in Table 2 are compared to those from calculations of the isolated sulfur atom according to four different sources: Cromer & Liberman^52^ (also available in the International Tables for Crystallography^53^), Henke et al.^54^, Sasaki (based on the Cromer & Liberman method)^55^, Brennan & Cowan^56^.

**Table 2:** Refined anomalous dispersion parameters *f*_*S*_*′* and *f*_*S*_*″* (unit *e*) for the YLID sulfur atom, compared to tabulated values for the isolated S atom.

Dataset	Refined *f*_*S*_*′*	Refined *f*_*S*_*″*	Temperature	Crystal
2	0.2694	0.6731	100 K	Natural shape 1
10	0.2587	0.6214	150 K	Natural shape 1
15	0.3189	0.5710	292 K	Test crystal 2
18	0.2865	0.5602	292 K	Natural shape 2
19	0.2196	0.5625	292 K	Test crystal 1
20	0.2667	0.5509	292 K	Natural shape 3
21	0.3681	0.5733	292 K	Test crystal 3
22	0.2861	0.5629	292 K	Test crystal 4
23	0.1930	0.5671	292 K	Test crystal 5
Average	0.27(5)	0.58(4)	–	–
Cromer & Liberman^52^	0.3331	0.5567	–	–
Henke et al.^54^	0.3354	0.5514	–	–
Sasaki^55^	0.328	0.590	–	–
Brennan & Cowan^56^	0.3338	0.5584	–	–

The refined values in Table 2 have a larger spread than the tabulated values. There is no clear trend in the refined values with respect to measurement parameters other than that the low-temperature results differ the most from the calculated values. *f*_*S*_*′* is significantly smaller and *f*_*S*_*″* is significantly larger than the calculated values. This trend relative to theory is, however, maintained for most datasets at 292 K to some degree, and the average value of *f*_*S*_*′* = 0.27(5) e is still significantly smaller than any of the tabulated theoretical values, outside a single standard deviation. The averaged value of *f*_*S*_*″* = 0.58(4) e is still larger than those of the tabulated values, except Sasaki, but within the standard deviation. We could speculate that this trend is due to the effect of chemical bonding within the YLID compound. However, it should be noted that we found that the anomalous dispersion parameters strongly correlate with the parameters of a ShelXL-type weighting scheme during HAR in olex2.refine, so that the refinement had to be done in a block-diagonal fashion (fixing one set of parameters while the other set is refined). The same holds for the extinction parameter. Hence, the reproducibility of the results presented depends on the refinement strategy.

### Chirality

**Fig. 3:**
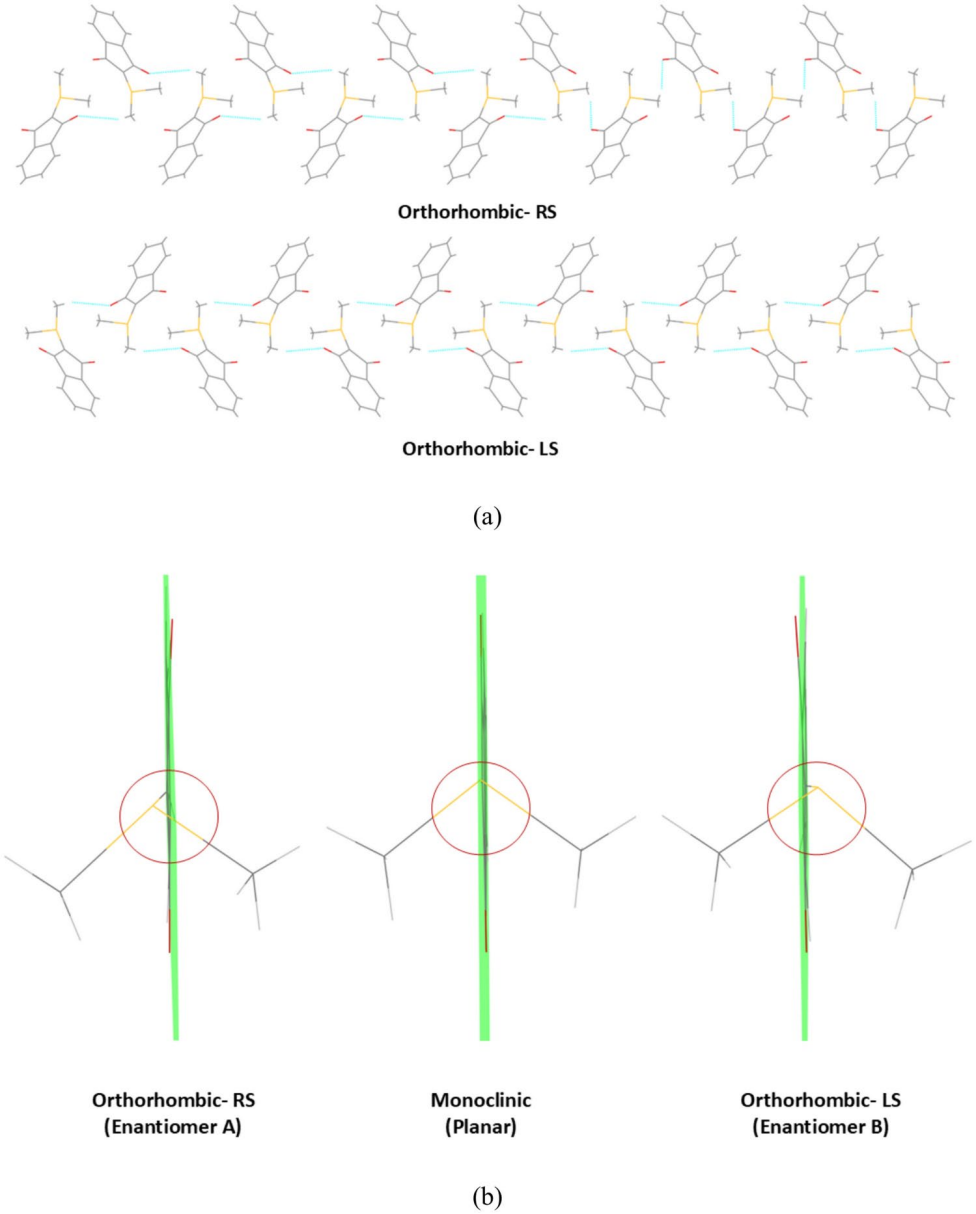
(**a**) Crystal packing down the *a* direction and parallel to the *b* direction showing the physical screw/ helical axis that we used to define the stereo descriptors *RS* (right-handed screw axis) and *LS* (left-handed screw axis). Here, test crystal 1 (*RS*) and test crystal 2 (*LS*) are shown. We note that the optical handedness and the orientation of the physical helical axis do not need to correspond, so we refrain from using descriptors *R* and *S* for the description of the overall handedness of the structure, but only refer to the geometric arrangement in the crystal packing here^57^. More details are given with respect to Figure S2 in the Supplementary Information. (**b**) Deviation of the C-S ylide bond and the C5-O2 bond from the indane plane in the *A* enantiomer (left) and the *B* enantiomer (right). The planar molecular geometry in the monoclinic polymorph is shown for comparison in the centre. The indane plane is represented with green colour. Yellow = sulfur, red = oxygen, grey = carbon, white = hydrogen.

Although the YLID molecule has no chiral centre and does not exhibit chirality in solution, the helical crystal packing of the orthorhombic polymorph (Fig. 3a) distorts the C-S ylide bond from the indane plane in different directions, creating two conformations which are mirror images of each other as they are locked in the solid state (Fig. 3b). This is a form of molecular planar chirality, however, because of the symmetry of the indane ring, priorities according to Cahn-Ingold-Prelog cannot be established and the stereo descriptors *R*_*p*_ and *S*_*p*_ are not applicable. We therefore refer to them simply as enantiomer *A* (occurs in the *RS* crystal form) and enantiomer *B* (occurs in the *LS* crystal form), based on the orientation of the substituents as shown in Fig. 3b. The torsion angles listed in Table S5 show that the sulfur atom is out of plane by about 7° with a distance of 0.28 Å perpendicular to the plane, whereas atom O2 is out of plane by about 4.5° on the other side. The effect of deviation from the indane plane magnifies for the two methyl groups on either side. The one on the side of the ylide S atom sticks out by about 19° more to this side of the plane compared to the other methyl group. For the monoclinic polymorph, this torsion angle difference for the methyl groups is only 2°.

We realized that we had no information on the chirality of the various spherical test crystals that we had obtained from the diffractometer vendors over a time span of about 25 years. All other crystallographers we asked were neither aware of the fact that the YLID test crystals are chiral nor of the chirality of the specimen that they own. Therefore, we concluded that the quantum-crystallographic protocol (QCP) that we suggest in reference^11^ should be extended by the determination of the absolute configuration of the crystal used. It is known that quantum-crystallographic models improve the refinement of Flack or related parameters used to determine the chirality because the phases are better determined in a physically more meaningful model.^51^ Therefore, we present the refined Flack parameter in the Hooft variant^58^ after HAR for the five different test crystals we own from various measurements in Table 3. The absolute configurations are all determined reliably, with a value close to zero. The standard uncertainty is on average 5 times larger for the Ag and Mo K_α_ measurements, however, it still allows an unambiguous determination of the chirality. We tested for all refinements that an inversion of the structure indeed led to the expected behaviour of switching the Hooft parameter from 0 to 1, accompanied by a small but significant increase of the R1 value.

**Table 3:** Flack parameter and chirality of test crystals after HAR.

Dataset	Test Crystal #	Enantiomer/Crystal Chirality	Hooft parameter	Wavelength
13	1	A/RS	0.009(13)	Ag = 0.56087 Å
14	1	A/RS	0.005(10)	Mo = 0.71073 Å
19	1	A/RS	0.004(2)	Cu = 1.54184 Å
15	2	B/LS	0.006(3)	Cu = 1.54184 Å
16	2	B/LS	-0.003(13)	Mo = 0.71073 Å
17	2	B/LS	0.004(14)	Ag = 0.56087 Å
21	3	B/LS	0.004(2)	Cu = 1.54184 Å
22	4	B/LS	0.004(2)	Cu = 1.54184 Å
23	5	A/RS	-0.001(1)	Cu = 1.54184 Å

### High-pressure modifications

The monoclinic polymorph is non-chiral, and the molecule is planar, i.e. the C-S and C-O bonds are in the indane plane (Fig. 3b, centre). According to reference 2, the orthorhombic polymorph (twisted molecular configuration) is more stable by 11.6 kJ/mol than the monoclinic form. In addition, here we calculated the energy difference between the two molecular forms in the isolated state as 11.7 kJ/mol at CCSD/def2-TZVP level, with the planar form being energetically more favourable. We note that the optimized geometry of the isolated molecule at the B3LYP/def2-TZVP level of theory is planar, too. This means that the energetic difference between planar and twisted modification is overcompensated by the crystal packing forces. In reference 2, the authors state that “the two polymorphs are monotropic and infinitely stable in the 100 − 298 K temperature range”, under ambient pressure. This prompted us to investigate whether a phase transition from orthorhombic (twisted) to monoclinic (planar) could be induced by external pressure. To this end, single-crystal X-ray diffraction measurements at high pressure were carried out at six different pressure points (0.12, 0.59, 2.12, 2.82, 3.23, 3.89 GPa) at the Swiss Light Source, Paul Scherrer Institute (see Materials and Methods for details). It turned out that there is no phase transition; the crystal form remains orthorhombic P2_1_2_1_2_1_, and the difference between the torsion angles involving the methyl groups remains constant at around 20°. Oxygen atom O2 is even pushed out of the plane slightly more than at ambient conditions. The *b*-axis in the orthorhombic form turns out to be the softest crystallographic direction during compression. Interestingly, the monoclinic phase exhibits Negative Thermal Expansion (NTE) along its *b*-direction while cooled to 100 K. However, due to the structural transformation, the *b*-axis in the orthorhombic form is not an equivalent direction to the monoclinic analogue, so there is no relationship between these phenomena.

**Fig. 4:**
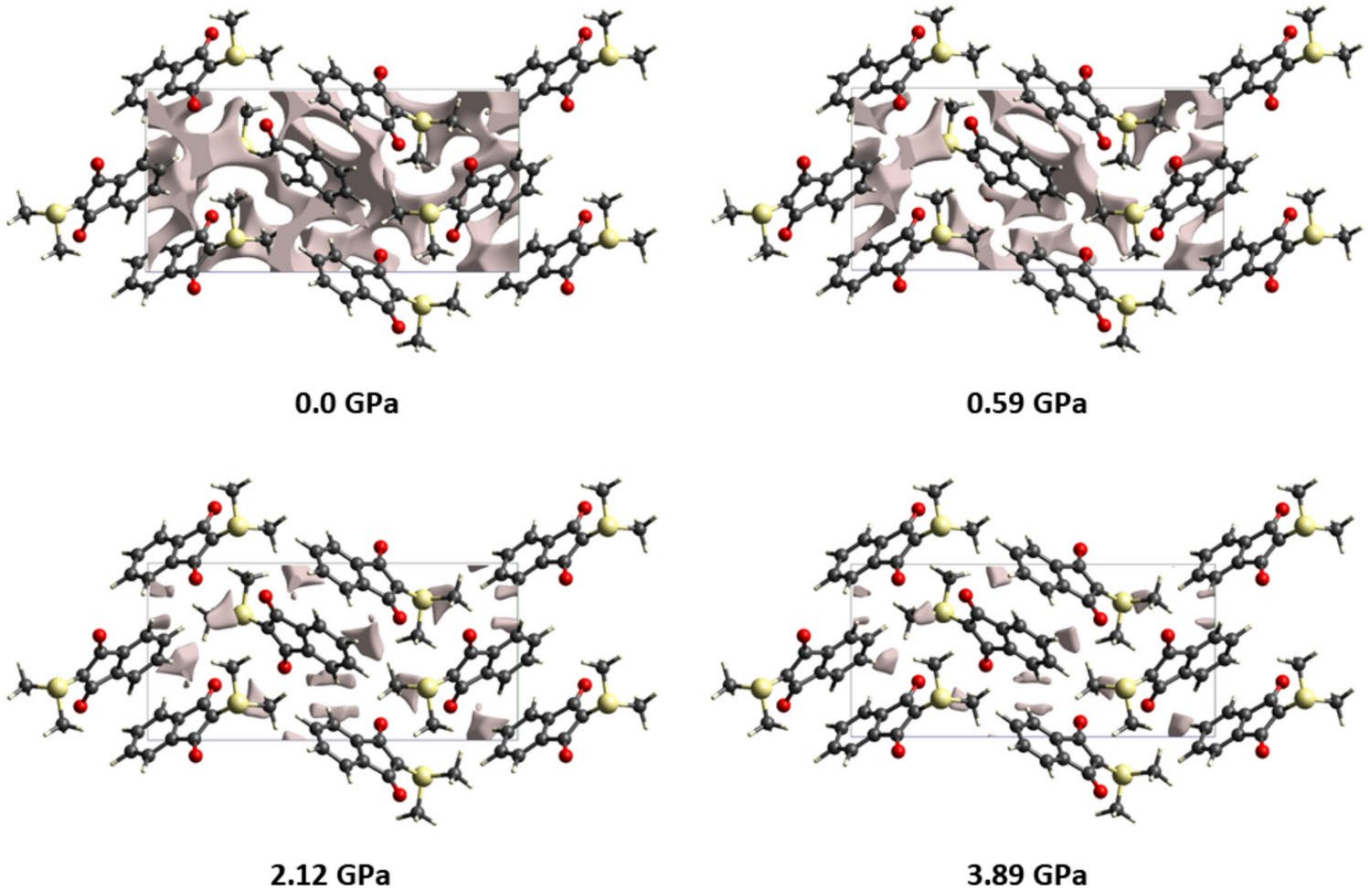
Electron-density isosurfaces at a value of 0.002 a.u. enclosing voids in the unit cells of the orthorhombic polymorph at different pressure points. At 0.0 GPa, void volume = 88.0 Å^3^, which encompasses 8.9% of the unit cell. At 0.59 GPa, 48.6 Å^3^ and 5.2%. At 2.21 GPa, 31.0 Å^3^ and 1.5%. At 3.89 GPa, 5.0 Å^3^ and 0.6%.

Although there is no correlation between any geometrical parameters and the pressure, there is a clear correlation of the lattice constants and the pressure, leading to a monotonously decreasing unit cell volume (Figure S3). The lattice constants *a*, *b*, and *c* decrease by a total of 6, 9, and 5%, and the volume decreases by 19%. Figure 4 shows that this regular, but large compression of the unit cell is accompanied by a loss of the void volumes from about 9 to 0.5% of the total unit cell volume^59^. This does influence the type and division of intermolecular interactions (see the Hirshfeld surface analysis below), but it does not influence the molecular geometry significantly.

### New YLID co-crystal phase

**Fig. 5:**
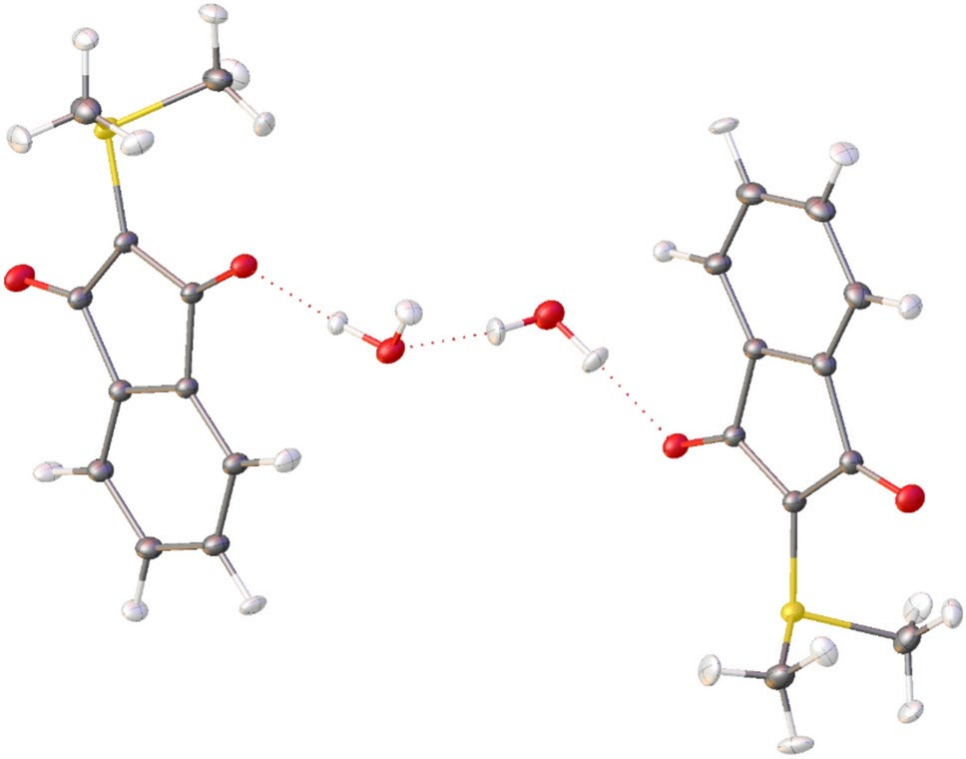
The crystal structure of the new YLID co-crystal phase involving two bridging water molecules. The YLID molecules are planar and the space group P2_1_/c is centrosymmetric.

Instead of external pressure, internal chemical pressure could also lead to a planarization of the YLID molecule starting from the chiral orthorhombic form. We used a crystal of natural shape and soaked it in wet paratone oil for approximately 7 months. This led to a single-crystal to single-crystal transformation, embedding two water molecules into the crystal structure (Fig. 5). The resulting crystal structure is indeed non-chiral, centrosymmetric (monoclinic space group P2_1_/c), and the two symmetry-independent YLID molecules are planar. The resulting crystal is twinned, but a HAR treatment could nevertheless successfully be performed including free refinement of the hydrogen-atom ADPs (Fig. 5). The observation of this new co-crystal means that hydrogen bonding with water breaks the helical structure, in favour of different intermolecular interactions, see the Hirshfeld surface analysis below. However, the reorientation of the molecules from twisted to planar does not break the crystallinity. This might have to do with the availability of about 9% of void volume in the structure, as discussed above.

### Hirshfeld surface analysis of all modifications and polymorphs

**Fig. 6:**
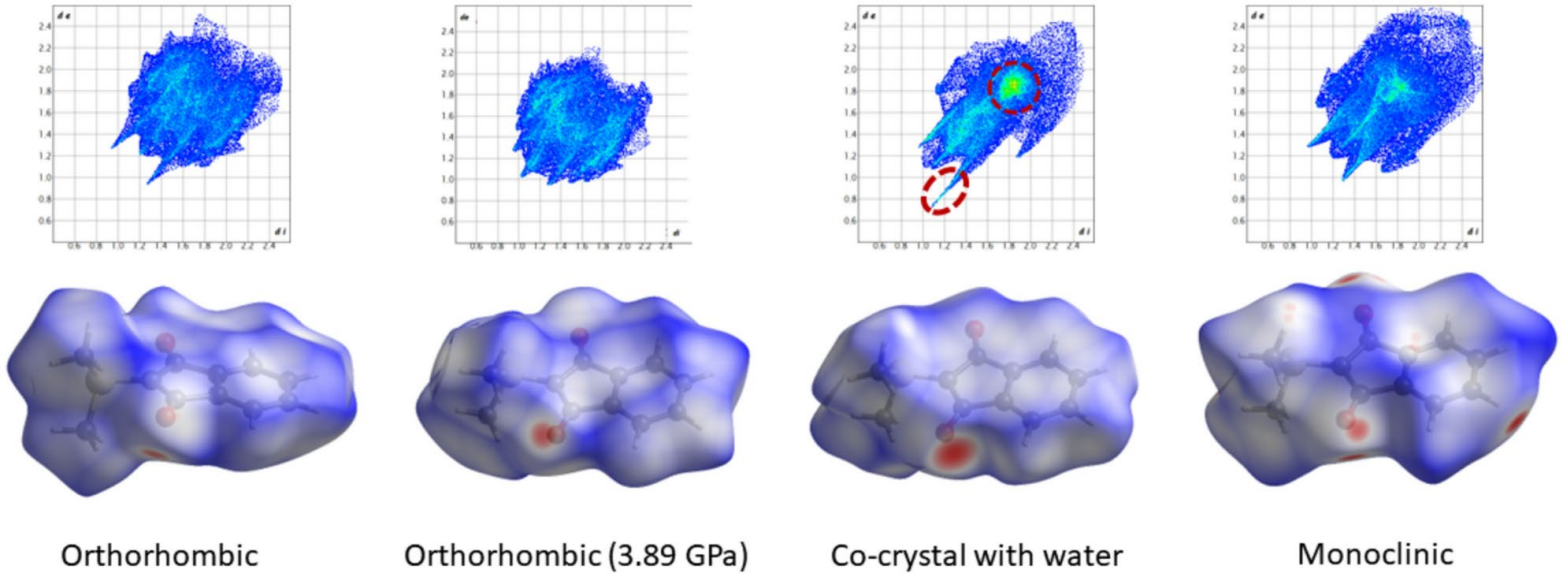
Hirshfeld surfaces and fingerprint plots for the YLID molecule in the different crystal packings. The red dashed circles in the fingerprint plot of the co-crystal structure represent the O…H (spike) and C…C (central marker) as mentioned in the text. The Hirshfeld surfaces are mapped with the property d_norm_ in a range from -0.289 a.u. (red) to 0 (white) to + 1.086 a.u. (blue). Results generated and depicted with CrystalExplorer^62^.

Hirshfeld surface analysis provides a qualitative tool to assess crystal packing and intermolecular interactions at one glance^60^. Red spots on the color-coded Hirshfeld surfaces (Fig. 6, second row) show regions of close intermolecular contacts, here associated with C-H…O hydrogen bonding as well as O–H…O hydrogen bonding in the case of the co-crystal with water. The shapes of the Hirshfeld surfaces, that represent the molecular van-der-Waals envelopes, are different in all four cases (orthorhombic, orthorhombic high-pressure, co-crystal with water, and monoclinic), illustrating different interactions with the environment. This is highlighted in the associated fingerprint plots^61^ (Fig. 6, first row), which have all different shapes and different features. In addition, Table S6 summarizes the percentage contribution of each contact type (C…C, C…H, O…H, and S…H) to the fingerprint plots.

The C-H…O hydrogen bonds can be observed as spikes in the fingerprint plots. The lower spike is longer (which means closer contacts) and more significant for the YLID co-crystal which is due to the O–H…O hydrogen bond with water. Whereas in the orthorhombic forms at ambient conditions and the monoclinic form, H…O contacts constitute 25% of all contacts, this number increases to 33% for the water co-crystal and drops to 22% for the high-pressure structure. For the latter, H…H and C-H...C(π) interactions become more important. H…H interactions are represented by the central spike in the fingerprint plots, which is longer than the H…O spike only for the high-pressure form. The compression necessarily leads to hydrogen atoms, as the outer skin of the molecules, coming in closer contact. This is corroborated by the fact that the diffuse region at long distances, which is an illustration of crystal voids, vanishes in the high-pressure form. The contribution of S…H hydrogen bonding is small with 4.5% across all structures.

C…C contacts representing π-π stacking interactions can be observed as a central marker in the fingerprint plots, especially pronounced in the co-crystal (red circle), where it amounts to 14% of all contacts. On the contrary, in the orthorhombic crystal packings, the contribution of π-π stacking is close to zero which is due to a zigzag arrangement of YLID molecules. The indane rings of the YLID molecules here interact mostly using C-H… π interaction, so that H…C contacts make about 30% of all contacts.

## Conclusions

We have shown in this work that there is much more to discover about and to learn from the YLID crystal structure, although it is the most common structure of all. This serves as a prominent example for the usefulness and capabilities of quantum-crystallographic refinement, and we encourage to revisit other fundamental compounds based on it. Hirshfeld atom refinement (HAR) provides accurate geometries, especially with respect to hydrogen atom positions, and X-ray wavefunction refinement (XWR) allows an experimental chemical bonding analysis, here confirming the charge-separated nature of the C-S ylide bond. Concerning anomalous dispersion of the sulfur atom, the refined* f*_*S*_*′* values are significantly smaller and *f*_*S*_*″* values larger than the tabulated values across all measurements under different conditions. This could be related to the effect of the chemical environment of the bonded atoms. The Flack parameter can be refined reliably and precisely within HAR, even for the long wavelengths used such as Ag Kα radiation.

We further discovered and explored the so far unknown chiral YLID crystal structure of left-handedness (*LS*). The chirality is an interplay between crystallographic helicity and molecular planar chirality, a complicated phenomenon for which we have not found an adequate description in the literature, although we believe that it is not rare. We confirmed earlier studies^2^ that showed that the chiral orthorhombic form (twisted molecule) does not undergo temperature-induced phase transition into the planar conformation in the monoclinic form. Therefore, we investigated high-pressure modifications to gain a different access to the phase diagram of YLID, but high pressure does not transform the twisted YLID molecules in the chiral orthorhombic form into the planar conformation in the monoclinic form either, it only reduces voids in the structure from 9% to 0.5% at *ca.* 4 GPa. However, chemical pressure does. Reaction of the orthorhombic crystals with residual moisture transforms them to a water co-crystal structure with planar YLID molecules that exhibits an entirely different intermolecular interaction pattern. This shows that the chemistry of YLID is much more versatile than it might seem from the perception of stable, hard, spherically ground test crystals we all have in our cupboards.

## Supplementary Information

Supplementary Information 1.

Supplementary Information 2.

Supplementary Information 3.

## Data Availability

The authors declare that the data supporting the findings of this study are available within the paper and its supplementary information file. Moreover, the crystallographic information files including lists of measured structure factors are available from the Cambridge Structural Database under deposition numbers CCDC-2309634, 2310173, 2321990–2321996. They can be obtained free of charge from https://www.ccdc.cam.ac.uk/structures.
