# Supplementary material for: Application of a quantum crystallographic protocol to YLID, the world's most common crystal structure
Source: Sci Rep. 2025 Apr 29;15:15045. doi: 10.1038/s41598-025-95269-3 (PMC12041244; doi:10.1038/s41598-025-95269-3)
Supplement: Supplementary file 1 — Supplementary Information 1. [file 41598_2025_95269_MOESM1_ESM.pdf]

Supplementary Information –

Application of a quantum crystallographic protocol to YLID, the world's most common crystal structure

Yaser Balmohammadi,[a] Lorraine A. Malaspina,[a] Yuiga Nakamura,[b] Georgia Cametti,[c] Michał Andrzejewski,[d] Milosz Siczek,[e] Simon Grabowsky[a,*]

[a] *University of Bern, Department of Chemistry, Biochemistry and Pharmaceutical Sciences, Freiestrasse 3, 3012 Bern, Switzerland.*

[b] *Japan Synchrotron Radiation Research Institute (JASRI), Sayo-cho, Hyogo 679-5198, Japan.*

[c] *University of Bern, Institute of Geological Sciences, Baltzerstrasse 3, 3012 Bern, Switzerland.*

[d] *Paul Scherrer Institute, Forschungsstrasse 111, 5232 Villigen, Switzerland.*

[e] *University of Wrocław, Faculty of Chemistry, F. Joliot-Curie 14, 50383 Wrocław, Poland.*

[*] *Correspondence e-mail: simon.grabowsky@unibe.ch*

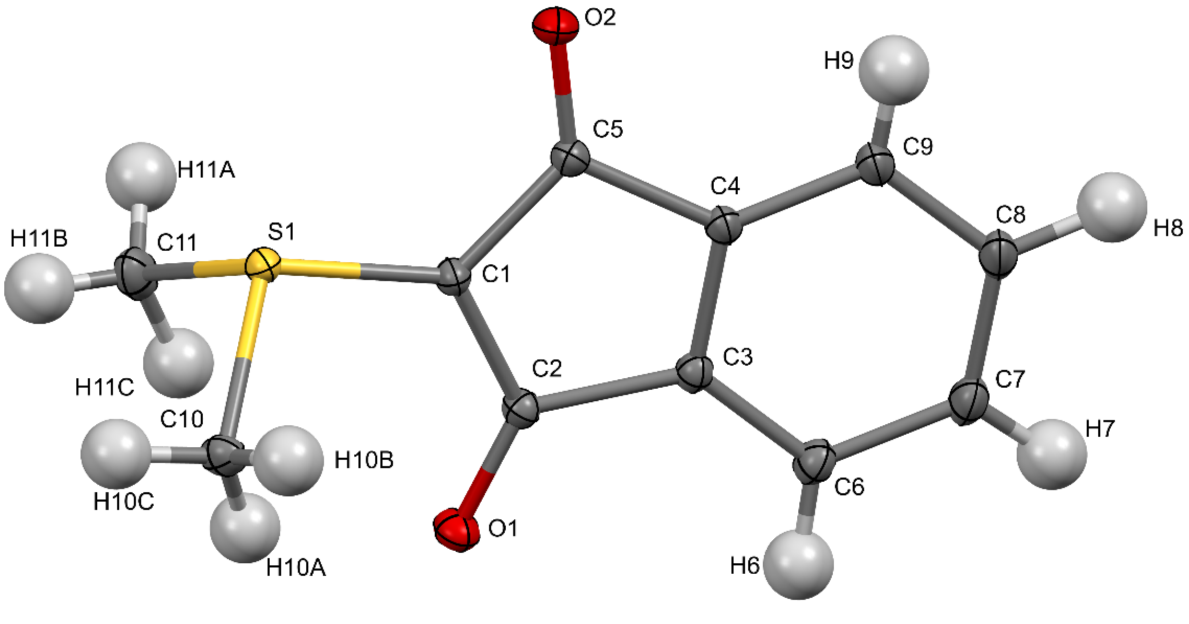

Figure S1. 3-D chemical structure of the YLID molecule with labeling scheme

Table S1. Summary of X-ray data collection conditions and refinement results of 23 repeated YLID measurements in the orthorhombic polymorph, from reference 1.

	Resolution (Å)/ CCDC-no.	Wavelength*	Temperature	Crystal/ Crystallographic Chirality	HAR/ XCW/ MM	R-value	Residual density e/Å^3 (max/min)
**1**	0.491/ 2309624	Ag	100K	Natural shape 1 LS§	yes/ yes/ yes	0.0118 0.0104 0.0110	0.110/-0.076 0.089/-0.072 0.090/-0.082
**2**	0.805/ 2309650	Cu	100K	Natural shape 1 LS	yes/ yes/ no	0.0100 0.0076 N/A	0.078/-0.053 0.052/-0.044 N/A
**3**	0.541/ 2309729	Mo**	100K	Natural shape 1 LS	yes/ yes/ yes	0.0157 0.0141 0.0147	0.119/-0.136 0.103/-0.116 0.121/-0.118
**4**	0.460/ 2310481	Mo#	100K	Natural shape 4 LS	yes/ yes / no	0.0078 0.0072 N/A	0.099/-0.065 0.101/-0.059 N/A
**5**	0.513/ 2310470	Mo#	100K	Natural shape 2 LS	yes/ yes / no	0.0100 0.0089 N/A	0.189/-0.072 0.165/-0.102 N/A
**6**	0.417/ 2310479	Synchrotron SPring-8	100K	Natural shape 5 LS	yes/ yes/ no	0.0088 0.0092 N/A	0.111/-0.104 0.065/-0.077 N/A
**7**	0.436/ 2310478	Synchrotron SPring-8	100K	Natural shape 6 LS	yes/ yes / no	0.0107 0.0101 N/A	0.113/-0.167 0.088/-0.103 N/A
**8**	0.491/ 2310480	Synchrotron SPring-8	100K	Natural shape 7 LS	yes/ yes / no	0.0157 0.0119 N/A	0.282/-0.516 0.184/-0.223 N/A
**9**	0.532/ 2309730	Ag	150K	Natural shape 1 LS	yes/ yes/ no	0.0110 0.0093 N/A	0.091/-0.059 0.075/-0.053 N/A
**10**	0.802/ 2309737	Cu	150K	Natural shape 1 LS	yes/ yes/ no	0.0070 0.0059 N/A	0.070/-0.046 0.051/-0.038 N/A
**11**	0.694/ 2309738	Ag	292K	Natural shape 2 LS	yes/ yes/ no	0.0141 0.0103 N/A	0.108/-0.052 0.064/-0.044 N/A
**12**	0.748/ CCDC-2309739	Mo**	292K	Natural shape 2 LS	yes/ yes/ no	0.0133 0.0109 N/A	0.069/-0.060 0.048/-0.055 N/A
**13**	0.623/ 2310190	Ag	292K	Test crystal 1 RS§	yes/ yes / no	0.0112 0.0096 N/A	0.089/-0.044 0.094/-0.090 N/A
**14**	0.620/	Mo**	292K	Test crystal 1 RS	yes/ yes /	0.0125 0.0115	0.075/-0.070 0.095/-0.097

	2310191				no	N/A	N/A
**15**	0.806/<br>2310482	Cu	292K	Test crystal 2<br>LS	yes/<br>yes/<br>no	0.0110<br>0.0062<br>N/A	0.070/-0.076<br>0.026/-0.029<br>N/A
**16**	0.657/<br>2310193	Mo**	292K	Test crystal 2<br>LS	yes/<br>yes/<br>yes	0.0164<br>0.0135<br>0.0141	0.087/-0.073<br>0.058/-0.058<br>0.066/-0.066
**17**	0.628/<br>2310192	Ag	292K	Test crystal 2<br>LS	yes/<br>yes/<br>yes	0.0178<br>0.0146<br>0.0143	0.145/-0.091<br>0.076/-0.069<br>0.072/-0.066
**18**	0.807/<br>2310197	Cu	292K	Natural shape 2<br>LS	yes/<br>yes /<br>no	0.0099<br>0.0088<br>N/A	0.048/-0.053<br>0.055/-0.073<br>N/A
**19**	0.789/<br>2310198	Cu	292K	Test crystal 1<br>RS	yes/<br>yes /<br>no	0.0120<br>0.0097<br>N/A	0.081/-0.050<br>0.105/-0.035<br>N/A
**20**	0.807/<br>2310199	Cu	292K	Natural shape 3<br>LS	yes/<br>yes /<br>no	0.0097<br>0.0083<br>N/A	0.059/-0.057<br>0.046/-0.059<br>N/A
**21**	0.787/<br>2310195	Cu	292K	Test crystal 3<br>LS	yes/<br>yes /<br>no	0.0125<br>0.0094<br>N/A	0.097/-0.095<br>0.059/-0.044<br>N/A
**22**	0.800/<br>2310196	Cu	292K	Test crystal 4<br>LS	yes/<br>yes /<br>no	0.0103<br>0.0099<br>N/A	0.059/-0.072<br>0.061/-0.067<br>N/A
**23**	0.784/<br>2310194	Cu	292K	Test crystal 5<br>RS	yes/<br>yes /<br>no	0.0108<br>0.0093<br>N/A	0.077/-0.053<br>0.057/-0.047<br>N/A

* Wavelength: Ag = 0.56087 Å; Mo = 0.71073 Å; Cu = 1.54184 Å; Synchrotron SPring8 = 0.2483 Å. # Synergy-R diffractometer. ** SuperNova diffractometer. § LS = left-handed screw axis; RS = right-handed screw axis.

Table S2. Crystallographic information, measurement details and IAM/HAR refinement results for datasets 24 and 25; details for 1-23 in reference 1. Dataset 24 is based on a non-merohedrally twinned crystal with fractional contributions of 84.8 and 15.2%.

**Experiment**	**Dataset 24**	**Dataset 25**
**Crystal**	New phase	Monoclinic polymorph
**Chemical formula**	$C_{22}H_{24}O_6S_2$	$C_{11}H_{10}O_2S$
**Form. weight (g/mol)**	448.53	206.267
**Crystal size (mm^3)**	0.17 x 0.09 x 0.06	0.21 x 0.28 x 0.35
**Crystal habit**	block	block
**Crystal color**	yellow	yellow
**Temperature (K)**	100	100
**Wavelength (Å)**	0.71073	1.54184
**a (Å)**	6.99850(10)	9.45647(4)
**b (Å)**	9.38690(10)	10.63614(5)
**c (Å)**	32.0797(2)	10.49215(6)
**α (°)**	90	90
**β (°)**	92.044(1)	104.9208(5)
**γ (°)**	90	90
**Volume (Å^3)**	2106.11(10)	1019.721(9)
**Z, Z'**	4,1	1, 0.25
**Space group**	P 2_1/c	P 2_1/c
**Number of refl.**	88189	82052
**Rint/Compl./Red.**	10.64%/99.7%/16.44	3.91%/100%/36.98
**Unique reflections**	23240	2218
**Unique observed [F>4sigma(F)]**	16688	2218
**Reflns theta min (°)**	3.342	4.840
**Reflns theta max (°)**	38.209	79.513
**Resolution (Å)**	0.575	0.784
**IAM:**		
**Number of parameters**	368	168
**R factor (obs)**	0.0369	0.0287
**R factor (all)**	0.0568	0.0287
**wR factor (obs)**	0.1197	0.0722
**Goodness of fit**	1.026	1.049
**Residual density max (e/Å^3)**	0.717	0.382
**Residual density min (e/Å^3)**	-0.639	-0.339
**HAR:**		
**Number of parameters**	537	242
**R factor (obs)**	0.0261	0.0151
**wR factor (obs)**	0.0645	0.0345
**Goodness of fit**	1.0288	1.0473
**Residual density max (e/Å^3)**	0.455	0.170
**Residual density min (e/Å^3)**	-0.253	-0.110

Table S3. Crystallographic, measurement, and refinement details (Independent Atom Model) for high-pressure YLID datasets

Experiment	HP_1	HP_2	HP_3
Chemical formula	$C_{11}H_{10}O_2S$	$C_{11}H_{10}O_2S$	$C_{11}H_{10}O_2S$
Form. weight (g/mol)	206.267	206.267	206.267
Pressure (GPa)	0	0.12	0.59
Crystal habit	block	block	block
Crystal color	yellow	yellow	yellow
Temperature (K)	293(2)	293(2)	293(2)
Wavelength (Å)	0.49225	0.49225	0.49225
a (Å)	5.96405(4)	5.93320(10)	5.83702(5)
b (Å)	9.03834(6)	8.9956(4)	8.8449(3)
c (Å)	18.39323(13)	18.3448(3)	18.1582(2)
α (º)	90	90	90
β (º)	90	90	90
γ (º)	90	90	90
Volume (Å^3)	991.490(12)	979.11(5)	937.47(4)
Z, Z’	4.1	4.1	4.1
Space group	$P\ 2_1\ 2_1\ 2_1$	$P\ 2_1\ 2_1\ 2_1$	$P\ 2_1\ 2_1\ 2_1$
R_{int}/Compl.	2.20%/99.3%	1.55%/56.4%	1.48%/59.2%
Reflns theta min (°)	1.739	2.499	2.539
Reflns theta max (°)	23.726	23.746	23.676
Resolution (Å)	0.610	0.610	0.610
No. parameters	130	129	129
R factor (obs)	3.21	2.85	3.02
wR factor (all)	9.28	8.05	8.87
Goodness of fit	1.076	1.072	1.081
**Experiment**	**HP_4**	**HP_5**	**HP_6**
Chemical formula	$C_{11}H_{10}O_2S$	$C_{11}H_{10}O_2S$	$C_{11}H_{10}O_2S$
Form. weight (g/mol)	206.267	206.267	206.267
Pressure (GPa)	2.12	2.82	3.23
Crystal habit	block	block	block
Crystal color	yellow	yellow	yellow
Temperature (K)	293(2)	293(2)	293(2)
Wavelength (Å)	0.49225	0.49225	0.49255
a (Å)	5.65690(10)	5.6332(2)	5.6203(3)
b (Å)	8.5142(6)	8.3667(15)	8.346(2)
c (Å)	17.8238(4)	17.6691(13)	17.5686(18)
α (º)	90	90	90
β (º)	90	90	90
γ (º)	90	90	90
Volume (Å^3)	858.47(7)	832.77(16)	824.1(2)
Z, Z’	4.1	4.1	4.1
Space group	$P\ 2_1\ 2_1\ 2_1$	$P\ 2_1\ 2_1\ 2_1$	$P\ 2_1\ 2_1\ 2_1$
R_{int}/Compl.	1.43%/57.4%	4.55%/57.3%	4.17%/56.6%
Reflns theta min (°)	2.616	2.628	2.637
Reflns theta max (°)	23.617	23.455	23.687
Resolution (Å)	0.610	0.620	0.610

**No. parameters**	129	129	129
**R factor (obs)**	4.43	8.79	9.80
**wR factor (all)**	13.64	23.64	25.74
**Goodness of fit**	1.173	1.066	1.135

**Experiment**	**HP_7**
**Chemical formula**	$C_{11}H_{10}O_2S$
**Form. weight (g/mol)**	206.267
**Pressure (GPa)**	3.89
**Crystal habit**	block
**Crystal color**	yellow
**Temperature (K)**	293(2)
**Wavelength (Å)**	0.49225
**a (Å)**	5.6136(5)
**b (Å)**	8.224(4)
**c (Å)**	17.427(4)
**α (°)**	90
**β (°)**	90
**γ (°)**	90
**Volume (Å^3)**	804.6(5)
**Z, Z'**	4.1
**Space group**	$P\ 2_1\ 2_1\ 2_1$
**R_{int}/Compl.**	4.80%/ 55.0%
**Reflns theta min (°)**	2.640
**Reflns theta max (°)**	23.705
**Resolution (Å)**	0.610
**No. parameters**	129
**R factor (obs)**	11.01
**wR factor (all)**	28.81
**Goodness of fit**	1.208

Table S4. C-H bond lengths from HAR, their average values and comparison to data from neutron diffraction as well as theoretical geometry optimization. Aromatic bonds: C6-H6 to C20-H20; methyl bonds: C10-H10A to C22-H22C; water: O3-H3A to O4-H4B. AVG: average value; SD: sample standard deviation. All values are in the unit Å. Neutron average bond length values for C-H (aromatic/methyl) and O-H (water) are extracted from Allen & Bruno[2] and Grabowsky et al.[3]. C-H(aromatic)= 1.083(17) Å; C-H(methyl)= 1.077(26) Å; O-H(water)= 0.971(6) Å.

**Dataset**	**24**		**25**		Geometry optimized	Geometry optimized (water-cocrystal)
	**Bond length (HAR)**	**Neutron minus HAR**	**Bond length (HAR)**	**Neutron minus HAR**	**Bond length**	**Bond length**
**C6-H6**	1.074	0.009	1.090	-0.007	1.082	1.082
**C7-H7**	1.079	0.004	1.093	-0.010	1.082	1.082
**C8-H8**	1.080	0.003	1.089	-0.006	1.082	1.082
**C9-H9**	1.081	0.002	1.062	0.021	1.082	1.082
**C17-H17**	1.081	0.002	-	-	-	1.082
**C18-H18**	1.065	0.018	-	-	-	1.081
**C19-H19**	1.095	-0.012	-	-	-	1.082
**C20-H20**	1.078	0.005	-	-	-	1.082
**C10-H10A**	1.077	0.000	1.074	0.003	1.088	1.088
**C10-H10B**	1.071	0.006	1.084	-0.007	1.087	1.087
**C10-H10C**	1.064	0.013	1.065	0.012	1.089	1.089
**C11-H11A**	1.075	0.002	1.090	-0.013	1.087	1.087
**C11-H11B**	1.062	0.015	1.079	-0.002	1.089	1.089
**C11-H11C**	1.095	-0.018	1.081	-0.004	1.088	1.088
**C21-H21A**	1.086	-0.009	-	-	-	1.087
**C21-H21B**	1.081	-0.004	-	-	-	1.088
**C21-H21C**	1.071	0.006	-	-	-	1.089
**C22-H22A**	1.079	-0.002	-	-	-	1.089
**C22-H22B**	1.068	0.009	-	-	-	1.087
**C22-H22C**	1.066	0.011	-	-	-	1.088
**O3-H3A**	0.976	-0.005	-	-	-	0.962
**O3-H3B**	0.944	0.027	-	-	-	0.961
**O4-H4A**	0.955	0.016	-	-	-	0.962
**O4-H4B**	0.960	0.011	-	-	-	0.963
**AVG (aromatic)**	1.079	0.004	1.083	-0.001	-	-
**AVG (methyl)**	1.074	0.002	1.078	-0.002	-	-
**AVG (water)**	0.958	0.012	-	-	-	-
**SD (aromatic)**	0.008	-	0.014	-	-	-
**SD (methyl)**	0.009	-	0.008	-	-	-
**SD (water)**	0.013	-	-	-	-	-

Table S5. A selection of torsion angles (°) and their difference. (*RS*) refers to test crystal 1 (dataset 19), whereas (*LS*) refers to test crystal 2 (dataset 15). HP = high pressure, at 3.89 GPa.

**Crystal structure**	**C10-S1-C1-C2**	**C11-S1-C1-C2**	**|Δangle|**	
**Orthorhombic (*RS*)**	62.5	-43.4	19.1	-
**Orthorhombic (*LS*)**	43.5	-62.1	18.6	-
**Orthorhombic (*LS*) HP**	43.1	-63.1	20.0	-
**Monoclinic**	51.8	-53.7	1.9	-
**Co-crystal with water***	49.7	-54.8	5.1	-
	51.4	-53.5	2.1	-
**Crystal structure**	**C4-C5-C1-S1**	**C3-C2-C1-S1**	**C9-C4-C5-O2**	**C6-C3-C2-O1**
**Orthorhombic (*RS*)**	-172.6	173.1	-4.6	0.8
**Orthorhombic (*LS*)**	172.6	-173.0	4.6	-0.6

*This form has two independent YLID molecules inside the asymmetric unit

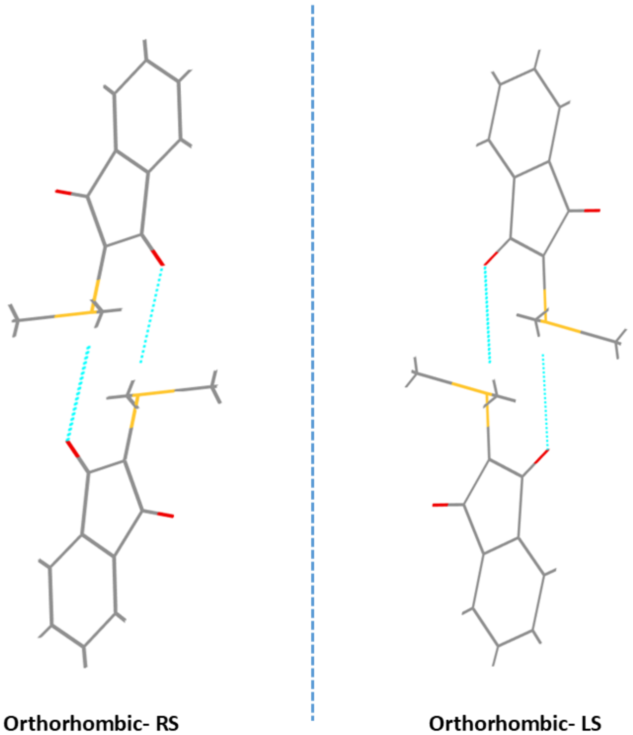

Figure S2. View down the *b* or -*b* directions with the helical axis that we used to define the stereo descriptors *RS* (right-handed screw) and *LS* (left-handed screw) in the centre of the ring spanned by the C-H…O hydrogen bonds. We chose oxygen atom O2 that is involved in the hydrogen bond to have a higher priority than atom O1 that is not involved in a hydrogen bond in the considered direction according to the Cahn-Ingold-Prelog rules. Otherwise, a definition of the sense of rotation would not have been possible. Since there are more crystallographic screw axes in the space group $P2_12_12_1$, our choice is ambiguous (in contrast to the textbook example quartz). However, the axis presented here and in Figure 3a is the most easily recognizable, and we refer to it as the physical screw axis that the molecules adopt via intermolecular hydrogen bonding. This, in turn, might be the reason for bending the sulfur atom out of the indane plane in a well-defined direction. Enantiomer *A* always coincides with a right-handed helix; enentiomer *B* always coincides with a left-handed helix.

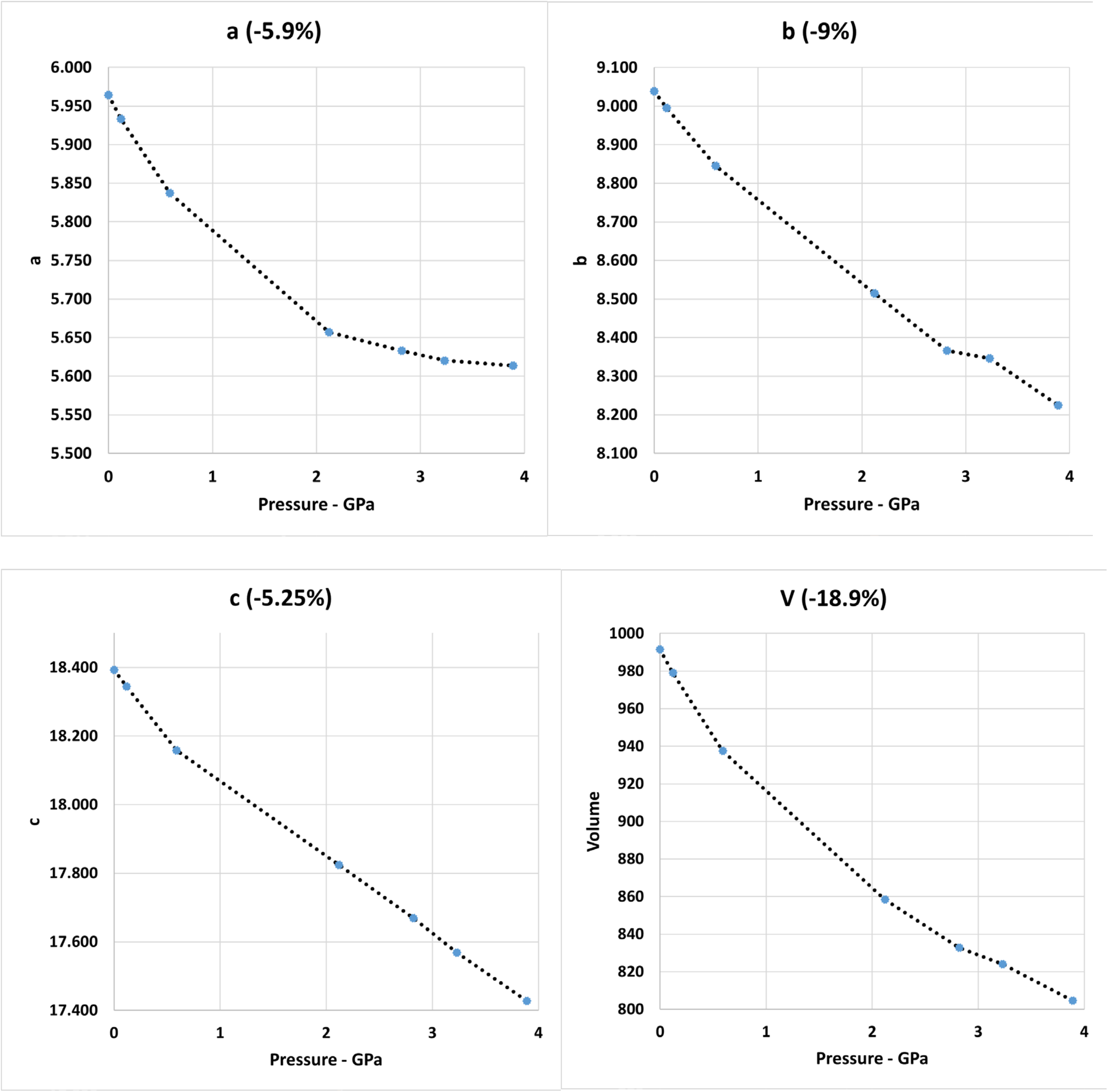

Figure S3. Evolution of lattice constants versus pressure in the 7 high-pressure data sets. The percentage shrinkage of the respective lattice constant/ volume is given in brackets. Units are Å and Å^3.

Table S6. Percentage contribution of O...H, S...H, C...C, and C...H intermolecular contacts of YLID molecules in different crystal packings according to the fingerprint plots shown in Figure 6.

	**Percentage of O…H**	**Percentage of S…H**	**Percentage of C…C**	**Percentage of C…H**
**Orthorhombic (*RS*) (dataset 19)**	24.6	4.6	0.2	27.4
**Orthorhombic (*LS*) (dataset 15)**	24.6	4.6	0.2	27.5
**Orthorhombic (*LS*) HP (3.89 GPa)**	22.4	4.6	0.4	31.7
**Monoclinic**	25.0	4.6	6.4	17.7
**Co-crystal with water**	32.9	4.6	14.3	6.0

References used in the Supplementary Information

[1] Balmohammadi, Y., Malaspina, L. A., Nakamura, Y., Cametti, G., Siczek, M. & Grabowsky, S. A quantum crystallographic protocol for general use. *Sci. Rep.* **15**, 13584 (2025).

[2] Allen, F. H. & Bruno, I. J. Bond lengths in organic and metal-organic compounds revisited: X—H bond lengths from neutron diffraction data. *Acta Cryst. B*. **66**, 380-386 (2010).

[3] Woińska, M., Grabowsky, S., Dominiak, P. M., Woźniak, K. & Jayatilaka, D. Hydrogen atoms can be located accurately and precisely by x-ray crystallography. *Sci. Adv.* **2**, e1600192 (2016).
