# Supplementary material for: Application of a quantum crystallographic protocol to YLID, the world's most common crystal structure
Source: Sci Rep. 2025 Apr 29;15:15045. doi: 10.1038/s41598-025-95269-3 (PMC12041244; doi:10.1038/s41598-025-95269-3)

checkCIF/PLATON report

Structure factors have been supplied for datablock(s) Ylid_0p00001, Ylid_0p12, Ylid_0p59, Ylid_2p12, Ylid_2p82, Ylid_3p23, Ylid_3p89

THIS REPORT IS FOR GUIDANCE ONLY. IF USED AS PART OF A REVIEW PROCEDURE FOR PUBLICATION, IT SHOULD NOT REPLACE THE EXPERTISE OF AN EXPERIENCED CRYSTALLOGRAPHIC REFEREE.

No syntax errors found. CIF dictionary Interpreting this report

Datablock: Ylid_0p00001

Bond precision:	C-C = 0.0021 A		Wavelength=0.49225
Cell:	a=5.96405(4)	b=9.03834(6)	c=18.39323(13)
	alpha=90	beta=90	gamma=90
Temperature:	293 K		

	Calculated	Reported
Volume	991.489(12)	991.490(12)
Space group	P 21 21 21	P 21 21 21
Hall group	P 2ac 2ab	P 2ac 2ab
Moiety formula	C11 H10 O2 S	C11 H10 O2 S
Sum formula	C11 H10 O2 S	C11 H10 O2 S
Mr	206.25	206.25
Dx,g cm-3	1.382	1.382
Z	4	4
Mu (mm-1)	0.115	0.114
F000	432.0	432.0
F000’	432.27	
h,k,lmax	9,14,30	8,12,25
Nref	4545[2604]	3249
Tmin,Tmax	0.989,0.994	0.836,1.000
Tmin’	0.986	

Correction method= # Reported T Limits: Tmin=0.836 Tmax=1.000
AbsCorr = MULTI-SCAN

Data completeness= 1.25/0.71 Theta(max)= 23.726

R(reflections)= 0.0306(3198) wR2(reflections)= 0.0868(3249)

S = 1.033 Npar= 130

The following ALERTS were generated. Each ALERT has the format
test-name_ALERT_alert-type_alert-level.
Click on the hyperlinks for more details of the test.

Alert level C

```
PLAT911_ALERT_3_C Missing FCF Refl Between Thmin & STh/L=    0.600          7 Report
                0  0  4,   0  0  6,   0  0  8,   0  0 18,   0  0 20,   1  0 21,
                0  0 22,
PLAT915_ALERT_3_C No Flack x Check Done: Low Friedel Pair Coverage         67 %
```

Alert level G

```
ABSMU01_ALERT_1_G  Calculation of _exptl_absorpt_correction_mu
                not performed for this radiation type.
PLAT012_ALERT_1_G N.O.K.   _shelx_res_checksum Found in CIF ......     Please Check
PLAT092_ALERT_4_G Check: Wavelength Given is not Cu,Ga,Mo,Ag,In Ka    0.49225 Ang.
PLAT142_ALERT_4_G s.u. on b - Axis Small or Missing .............    0.00006 Ang.
PLAT143_ALERT_4_G s.u. on c - Axis Small or Missing .............    0.00013 Ang.
PLAT199_ALERT_1_G Reported _cell_measurement_temperature ..... (K)        293 Check
PLAT200_ALERT_1_G Reported   _diffrn_ambient_temperature ..... (K)        293 Check
PLAT910_ALERT_3_G Missing # of FCF Reflection(s) Below Theta(Min).          1 Note
                0  0  2,
PLAT912_ALERT_4_G Missing # of FCF Reflections Above STh/L=  0.600        399 Note
PLAT933_ALERT_2_G Number of HKL-OMIT Records in Embedded .res File          2 Note
               -1  1 22,  -1  1  2,
PLAT941_ALERT_3_G Average HKL Measurement Multiplicity ..........         4.2 Low
PLAT951_ALERT_5_G Calculated (ThMax) and CIF-Reported Kmax Differ           2 Units
PLAT952_ALERT_5_G Calculated (ThMax) and CIF-Reported Lmax Differ.          5 Units
PLAT957_ALERT_1_G Calculated (ThMax) and Actual (FCF) Kmax Differ           2 Units
PLAT958_ALERT_1_G Calculated (ThMax) and Actual (FCF) Lmax Differ.          5 Units
PLAT978_ALERT_2_G Number C-C Bonds with Positive Residual Density.          5 Info
PLAT984_ALERT_1_G The  C-f'=   -0.0004 Deviates from the B&C-Value     0.0006 Check
PLAT984_ALERT_1_G The  O-f'=    0.0008 Deviates from the B&C-Value     0.0038 Check
PLAT984_ALERT_1_G The  S-f'=    0.0506 Deviates from the B&C-Value     0.0646 Check
```

0 **ALERT level A** = Most likely a serious problem - resolve or explain
0 **ALERT level B** = A potentially serious problem, consider carefully
2 **ALERT level C** = Check. Ensure it is not caused by an omission or oversight
19 **ALERT level G** = General information/check it is not something unexpected

9 ALERT type 1 CIF construction/syntax error, inconsistent or missing data
2 ALERT type 2 Indicator that the structure model may be wrong or deficient
4 ALERT type 3 Indicator that the structure quality may be low
4 ALERT type 4 Improvement, methodology, query or suggestion
2 ALERT type 5 Informative message, check

Datablock: Ylid_0p12

Bond precision: C-C = 0.0038 A   Wavelength=0.49225

Cell:	a=5.9332(1)	b=8.9956(4)	c=18.3448(3)
	alpha=90	beta=90	gamma=90
Temperature:	293 K		

	Calculated	Reported
Volume	979.11(5)	979.11(5)
Space group	P 21 21 21	P 21 21 21
Hall group	P 2ac 2ab	P 2ac 2ab
Moiety formula	C11 H10 O2 S	C11 H10 O2 S
Sum formula	C11 H10 O2 S	C11 H10 O2 S
Mr	206.25	206.25
Dx,g cm-3	1.399	1.399
Z	4	4
Mu (mm-1)	0.116	0.116
F000	432.0	432.0
F000'	432.27	
h,k,lmax	9,14,30	8,8,25
Nref	4492[2576]	1559
Tmin,Tmax	0.989,0.994	0.848,1.000
Tmin'	0.986	

Correction method= # Reported T Limits: Tmin=0.848 Tmax=1.000
AbsCorr = MULTI-SCAN

Data completeness= 0.61/0.35 Theta(max)= 23.746

R(reflections)= 0.0285(1475) wR2(reflections)= 0.0805(1559)

S = 1.072 Npar= 129

The following ALERTS were generated. Each ALERT has the format
test-name_ALERT_alert-type_alert-level.
Click on the hyperlinks for more details of the test.

Alert level A

PLAT029_ALERT_3_A _diffrn_measured_fraction_theta_full value Low . 0.564 Why?

Author Response: Our experiment was done using a diamond-anvil cell rotated about one axis by 76 deg. Hence, due to the strategy and a limited access to the sample in a high-pressure cell no complete data was collected.

Alert level B

PLAT911_ALERT_3_B Missing FCF Refl Between Thmin & STh/L= 0.600 392 Report

```
                0  2  0,   1  2  0,   1  3  0,   2  3  0,   0  4  0,   1  4  0,
                2  4  0,   3  4  0,   4  4  0,   1  5  0,   2  5  0,   3  5  0,
                4  5  0,   5  5  0,   6  5  0,   0  6  0,   1  6  0,   2  6  0,
                3  6  0,   4  6  0,   5  6  0,   1  7  0,   2  7  0,   3  7  0,
                4  7  0,   5  7  0,   0  8  0,   1  8  0,   2  8  0,   3  8  0,
                4  8  0,   0  2  1,   1  2  1,   0  3  1,   1  3  1,   2  3  1,
                0  4  1,   1  4  1,   2  4  1,   3  4  1,   0  5  1,   1  5  1,
                2  5  1,   3  5  1,   4  5  1,   5  5  1,   6  5  1,   0  6  1,
                1  6  1,   2  6  1,   3  6  1,   4  6  1,   5  6  1,   0  7  1,
                1  7  1,   2  7  1,   3  7  1,   4  7  1,   5  7  1,   0  8  1,
                1  8  1,   2  8  1,   3  8  1,   4  8  1,   0  2  2,   1  2  2,
                0  3  2,   1  3  2,   2  3  2,   0  4  2,   1  4  2,   2  4  2,
                3  4  2,   0  5  2,   1  5  2,   2  5  2,   3  5  2,   4  5  2,
                5  5  2,   0  6  2,   1  6  2,   2  6  2,   3  6  2,   4  6  2,
                5  6  2,   0  7  2,   1  7  2,   2  7  2,   3  7  2,   4  7  2,
                5  7  2,   0  8  2,   1  8  2,   2  8  2,   3  8  2,   4  8  2,
PLAT915_ALERT_3_B No Flack x Check Done: Low Friedel Pair Coverage         27 %
```

Alert level G

```
ABSMU01_ALERT_1_G  Calculation of _exptl_absorpt_correction_mu
                not performed for this radiation type.
PLAT092_ALERT_4_G Check: Wavelength Given is not Cu,Ga,Mo,Ag,In Ka    0.49225 Ang.
PLAT199_ALERT_1_G Reported _cell_measurement_temperature ..... (K)        293 Check
PLAT200_ALERT_1_G Reported   _diffrn_ambient_temperature ..... (K)        293 Check
PLAT910_ALERT_3_G Missing # of FCF Reflection(s) Below Theta(Min).          3 Note
                0  1  1,   0  0  2,   0  1  2,
PLAT912_ALERT_4_G Missing # of FCF Reflections Above STh/L=  0.600        411 Note
PLAT913_ALERT_3_G Missing # of Very Strong Reflections in FCF ....          1 Note
                0  2  2,
PLAT941_ALERT_3_G Average HKL Measurement Multiplicity ..........         4.9 Low
PLAT951_ALERT_5_G Calculated (ThMax) and CIF-Reported Kmax Differ           6 Units
PLAT952_ALERT_5_G Calculated (ThMax) and CIF-Reported Lmax Differ.          5 Units
PLAT957_ALERT_1_G Calculated (ThMax) and Actual (FCF) Kmax Differ           6 Units
PLAT958_ALERT_1_G Calculated (ThMax) and Actual (FCF) Lmax Differ.          5 Units
PLAT978_ALERT_2_G Number C-C Bonds with Positive Residual Density.          2 Info
PLAT984_ALERT_1_G The  C-f'=   -0.0004 Deviates from the B&C-Value     0.0006 Check
PLAT984_ALERT_1_G The  O-f'=    0.0008 Deviates from the B&C-Value     0.0038 Check
PLAT984_ALERT_1_G The  S-f'=    0.0506 Deviates from the B&C-Value     0.0646 Check
```

---

1 **ALERT level A** = Most likely a serious problem - resolve or explain
2 **ALERT level B** = A potentially serious problem, consider carefully
0 **ALERT level C** = Check. Ensure it is not caused by an omission or oversight
16 **ALERT level G** = General information/check it is not something unexpected

8 ALERT type 1 CIF construction/syntax error, inconsistent or missing data
1 ALERT type 2 Indicator that the structure model may be wrong or deficient
6 ALERT type 3 Indicator that the structure quality may be low
2 ALERT type 4 Improvement, methodology, query or suggestion
2 ALERT type 5 Informative message, check

Datablock: Ylid_0p59

```
Bond precision:   C-C = 0.0038 A          Wavelength=0.49225

Cell:              a=5.83702(5)      b=8.8449(3)      c=18.1582(2)
                   alpha=90          beta=90          gamma=90
Temperature:       293 K
```

	Calculated	Reported
Volume	937.47(3)	937.47(4)
Space group	P 21 21 21	P 21 21 21
Hall group	P 2ac 2ab	P 2ac 2ab
Moiety formula	C11 H10 O2 S	C11 H10 O2 S
Sum formula	C11 H10 O2 S	C11 H10 O2 S
Mr	206.25	206.25
Dx,g cm-3	1.461	1.461
Z	4	4
Mu (mm-1)	0.121	0.121
F000	432.0	432.0
F000’	432.27	
h,k,lmax	9,14,29	8,8,25
Nref	4274[2457]	1543
Tmin,Tmax	0.988,0.994	0.871,1.000
Tmin’	0.986	

```
Correction method= # Reported T Limits: Tmin=0.871 Tmax=1.000
AbsCorr = MULTI-SCAN

Data completeness= 0.63/0.36        Theta(max)= 23.676

R(reflections)= 0.0304( 1466)                      wR2(reflections)=
                                                   0.0828( 1543)
S = 1.006                  Npar= 129
```

The following ALERTS were generated. Each ALERT has the format
test-name_ALERT_alert-type_alert-level.
Click on the hyperlinks for more details of the test.

Alert level A

PLAT029_ALERT_3_A _diffrn_measured_fraction_theta_full value Low .   0.592 Why?

Author Response: Our experiment was done using a diamond-anvil cell rotated about one axis by 76 deg. Hence, due to the strategy and a limited access to the sample in a high-pressure cell no complete data was collected.

Alert level B

```
PLAT911_ALERT_3_B Missing FCF Refl Between Thmin & STh/L=    0.600        350 Report
                0  2  0,   1  2  0,   1  3  0,   2  3  0,   3  3  0,   0  4  0,
                1  4  0,   2  4  0,   3  4  0,   4  4  0,   1  5  0,   2  5  0,
                3  5  0,   4  5  0,   5  5  0,   6  5  0,   0  6  0,   1  6  0,
                2  6  0,   3  6  0,   4  6  0,   5  6  0,   1  7  0,   2  7  0,
                3  7  0,   4  7  0,   5  7  0,   0  8  0,   1  8  0,   2  8  0,
                3  8  0,   4  8  0,   0  2  1,   1  2  1,   0  3  1,   1  3  1,
                2  3  1,   0  4  1,   1  4  1,   2  4  1,   3  4  1,   4  4  1,
                0  5  1,   1  5  1,   2  5  1,   3  5  1,   4  5  1,   5  5  1,
                6  5  1,   0  6  1,   1  6  1,   2  6  1,   3  6  1,   4  6  1,
                5  6  1,   0  7  1,   1  7  1,   2  7  1,   3  7  1,   4  7  1,
                5  7  1,   0  8  1,   1  8  1,   2  8  1,   3  8  1,   4  8  1,
                0  2  2,   0  3  2,   1  3  2,   2  3  2,   0  4  2,   1  4  2,
                2  4  2,   3  4  2,   0  5  2,   1  5  2,   2  5  2,   3  5  2,
                4  5  2,   5  5  2,   6  5  2,   0  6  2,   1  6  2,   2  6  2,
                3  6  2,   4  6  2,   5  6  2,   0  7  2,   1  7  2,   2  7  2,
                3  7  2,   4  7  2,   5  7  2,   0  8  2,   1  8  2,   2  8  2,
PLAT915_ALERT_3_B No Flack x Check Done: Low Friedel Pair Coverage         28 %
```

Alert level G

```
ABSMU01_ALERT_1_G  Calculation of _exptl_absorpt_correction_mu
                not performed for this radiation type.
PLAT092_ALERT_4_G Check: Wavelength Given is not Cu,Ga,Mo,Ag,In Ka    0.49225 Ang.
PLAT199_ALERT_1_G Reported _cell_measurement_temperature ..... (K)        293 Check
PLAT200_ALERT_1_G Reported   _diffrn_ambient_temperature ..... (K)        293 Check
PLAT910_ALERT_3_G Missing # of FCF Reflection(s) Below Theta(Min).          3 Note
                0  1  1,   0  0  2,   0  1  2,
PLAT912_ALERT_4_G Missing # of FCF Reflections Above STh/L=  0.600        411 Note
PLAT913_ALERT_3_G Missing # of Very Strong Reflections in FCF ....          1 Note
                0  2  2,
PLAT933_ALERT_2_G Number of HKL-OMIT Records in Embedded .res File          2 Note
                8  2  2,   0  2  8,
PLAT941_ALERT_3_G Average HKL Measurement Multiplicity ...........        4.6 Low
PLAT951_ALERT_5_G Calculated (ThMax) and CIF-Reported Kmax Differ           6 Units
PLAT952_ALERT_5_G Calculated (ThMax) and CIF-Reported Lmax Differ.          4 Units
PLAT957_ALERT_1_G Calculated (ThMax) and Actual (FCF) Kmax Differ           6 Units
PLAT958_ALERT_1_G Calculated (ThMax) and Actual (FCF) Lmax Differ.          4 Units
PLAT978_ALERT_2_G Number C-C Bonds with Positive Residual Density.          1 Info
PLAT984_ALERT_1_G The  C-f'=   -0.0004 Deviates from the B&C-Value     0.0006 Check
PLAT984_ALERT_1_G The  O-f'=    0.0008 Deviates from the B&C-Value     0.0038 Check
PLAT984_ALERT_1_G The  S-f'=    0.0506 Deviates from the B&C-Value     0.0646 Check
```

1 **ALERT level A** = Most likely a serious problem - resolve or explain
2 **ALERT level B** = A potentially serious problem, consider carefully
0 **ALERT level C** = Check. Ensure it is not caused by an omission or oversight
17 **ALERT level G** = General information/check it is not something unexpected

8 ALERT type 1 CIF construction/syntax error, inconsistent or missing data
2 ALERT type 2 Indicator that the structure model may be wrong or deficient
6 ALERT type 3 Indicator that the structure quality may be low
2 ALERT type 4 Improvement, methodology, query or suggestion
2 ALERT type 5 Informative message, check

Datablock: Ylid_2p12

Bond precision: C-C = 0.0061 A Wavelength=0.49225

Cell:	a=5.65687(7)	b=8.5142(6)	c=17.8238(4)
	alpha=90	beta=90	gamma=90
Temperature:	293 K		

	Calculated	Reported
Volume	858.46(6)	858.46(6)
Space group	P 21 21 21	P 21 21 21
Hall group	P 2ac 2ab	P 2ac 2ab
Moiety formula	C11 H10 O2 S	C11 H10 O2 S
Sum formula	C11 H10 O2 S	C11 H10 O2 S
Mr	206.25	206.25
Dx,g cm-3	1.596	1.596
Z	4	4
Mu (mm-1)	0.133	0.132
F000	432.0	432.0
F000'	432.27	
h,k,lmax	9,13,29	8,7,24
Nref	3869[2235]	1314
Tmin,Tmax	0.987,0.993	0.559,1.000
Tmin'	0.984	

Correction method= # Reported T Limits: Tmin=0.559 Tmax=1.000
AbsCorr = MULTI-SCAN

Data completeness= 0.59/0.34 Theta(max)= 23.617

R(reflections)= 0.0441(1246) wR2(reflections)= 0.1337(1314)

S = 1.151 Npar= 129

The following ALERTS were generated. Each ALERT has the format
test-name_ALERT_alert-type_alert-level.
Click on the hyperlinks for more details of the test.

Alert level A

PLAT029_ALERT_3_A _diffrn_measured_fraction_theta_full value Low . 0.574 Why?

Author Response: Our experiment was done using a diamond-anvil cell rotated about one axis by 76 deg. Hence, due to the strategy and a limited access to the sample in a high-pressure cell no complete data was collected.

Alert level B

```
PLAT911_ALERT_3_B Missing FCF Refl Between Thmin & STh/L=    0.600        303 Report
                0  2  0,   0  4  0,   0  6  0,   1  2  0,   1  3  0,   1  4  0,
                1  5  0,   1  6  0,   1  7  0,   2  3  0,   2  4  0,   2  5  0,
                2  6  0,   2  7  0,   3  3  0,   3  4  0,   3  5  0,   3  6  0,
                3  7  0,   4  4  0,   4  5  0,   4  6  0,   4  7  0,   5  5  0,
                5  6  0,   0  2  1,   0  3  1,   0  4  1,   0  5  1,   0  6  1,
                0  7  1,   1  2  1,   1  3  1,   1  4  1,   1  5  1,   1  6  1,
                1  7  1,   2  3  1,   2  4  1,   2  5  1,   2  6  1,   2  7  1,
                3  4  1,   3  5  1,   3  6  1,   3  7  1,   4  4  1,   4  5  1,
                4  6  1,   4  7  1,   5  5  1,   5  6  1,   0  2  2,   0  3  2,
                0  4  2,   0  5  2,   0  6  2,   0  7  2,   1  2  2,   1  3  2,
                1  4  2,   1  5  2,   1  6  2,   1  7  2,   2  3  2,   2  4  2,
                2  5  2,   2  6  2,   2  7  2,   3  4  2,   3  5  2,   3  6  2,
                3  7  2,   4  5  2,   4  6  2,   4  7  2,   5  5  2,   5  6  2,
                0  1  3,   0  2  3,   0  3  3,   0  4  3,   0  5  3,   0  6  3,
                0  7  3,   1  2  3,   1  3  3,   1  4  3,   1  5  3,   1  6  3,
                1  7  3,   2  3  3,   2  4  3,   2  5  3,   2  6  3,   2  7  3,
PLAT915_ALERT_3_B No Flack x Check Done: Low Friedel Pair Coverage         26 %
```

Alert level C

```
PLAT089_ALERT_3_C Poor Data / Parameter Ratio (Zmax < 18) ........       6.89 Note
PLAT340_ALERT_3_C Low Bond Precision on  C-C Bonds ..............     0.0061 Ang.
```

Alert level G

```
ABSMU01_ALERT_1_G  Calculation of _exptl_absorpt_correction_mu
                not performed for this radiation type.
PLAT032_ALERT_4_G Std. Uncertainty on Flack Parameter Value High .      0.300 Report
PLAT072_ALERT_2_G SHELXL First  Parameter in WGHT  Unusually Large       0.11 Report
PLAT092_ALERT_4_G Check: Wavelength Given is not Cu,Ga,Mo,Ag,In Ka    0.49225 Ang.
PLAT199_ALERT_1_G Reported _cell_measurement_temperature ..... (K)        293 Check
PLAT200_ALERT_1_G Reported   _diffrn_ambient_temperature ..... (K)        293 Check
PLAT850_ALERT_4_G Check Flack Parameter Exact Value 0.00 with s.u.       0.30 Check
PLAT910_ALERT_3_G Missing # of FCF Reflection(s) Below Theta(Min).          3 Note
                0  1  1,   0  0  2,   0  1  2,
PLAT912_ALERT_4_G Missing # of FCF Reflections Above STh/L=  0.600        349 Note
PLAT913_ALERT_3_G Missing # of Very Strong Reflections in FCF ....          1 Note
                0  2  2,
PLAT933_ALERT_2_G Number of HKL-OMIT Records in Embedded .res File          2 Note
                2  1 19,   2  1 18,
PLAT951_ALERT_5_G Calculated (ThMax) and CIF-Reported Kmax Differ           6 Units
PLAT952_ALERT_5_G Calculated (ThMax) and CIF-Reported Lmax Differ.          5 Units
PLAT957_ALERT_1_G Calculated (ThMax) and Actual (FCF) Kmax Differ           6 Units
PLAT958_ALERT_1_G Calculated (ThMax) and Actual (FCF) Lmax Differ.          5 Units
PLAT978_ALERT_2_G Number C-C Bonds with Positive Residual Density.         10 Info
PLAT984_ALERT_1_G The  C-f'=   -0.0004 Deviates from the B&C-Value     0.0006 Check
PLAT984_ALERT_1_G The  O-f'=    0.0008 Deviates from the B&C-Value     0.0038 Check
```

```
PLAT984_ALERT_1_G The  S-f'=    0.0506 Deviates from the B&C-Value     0.0646 Check
```

```
   1 ALERT level A = Most likely a serious problem - resolve or explain
   2 ALERT level B = A potentially serious problem, consider carefully
   2 ALERT level C = Check. Ensure it is not caused by an omission or oversight
  19 ALERT level G = General information/check it is not something unexpected

   8 ALERT type 1 CIF construction/syntax error, inconsistent or missing data
   3 ALERT type 2 Indicator that the structure model may be wrong or deficient
   7 ALERT type 3 Indicator that the structure quality may be low
   4 ALERT type 4 Improvement, methodology, query or suggestion
   2 ALERT type 5 Informative message, check
```

Datablock: Ylid_2p82

```
Bond precision:   C-C = 0.0125 A           Wavelength=0.49225

Cell:              a=5.6326(2)     b=8.3700(15)      c=17.6601(13)
                   alpha=90        beta=90           gamma=90
Temperature:       293 K

                  Calculated                    Reported
Volume            832.58(16)                    832.58(16)
Space group       P 21 21 21                    P 21 21 21
Hall group        P 2ac 2ab                     P 2ac 2ab
Moiety formula    C11 H10 O2 S                  C11 H10 O2 S
Sum formula       C11 H10 O2 S                  C11 H10 O2 S
Mr                206.25                        206.25
Dx,g cm-3         1.645                         1.645
Z                 4                             4
Mu (mm-1)         0.137                         0.136
F000              432.0                         432.0
F000'             432.27
h,k,lmax          9,13,28                       8,7,24
Nref              3696[ 2138]                   1284
Tmin,Tmax         0.987,0.993                   0.239,1.000
Tmin'             0.984

Correction method= # Reported T Limits: Tmin=0.239 Tmax=1.000
AbsCorr = MULTI-SCAN

Data completeness= 0.60/0.35       Theta(max)= 23.460
```

R(reflections)= 0.0893(1122)

wR2(reflections)= 0.2478(1284)

S = 1.130

Npar= 129

The following ALERTS were generated. Each ALERT has the format
test-name_ALERT_alert-type_alert-level.
Click on the hyperlinks for more details of the test.

Alert level A

PLAT029_ALERT_3_A _diffrn_measured_fraction_theta_full value Low . 0.573 Why?

Author Response: Our experiment was done using a diamond-anvil cell rotated about one axis by 76 deg. Hence, due to the strategy and a limited access to the sample in a high-pressure cell no complete data was collected.

Alert level B

PLAT340_ALERT_3_B Low Bond Precision on C-C Bonds .... 0.0125 Ang.
PLAT911_ALERT_3_B Missing FCF Refl Between Thmin & STh/L= 0.600 304 Report

0 2 0,	0 4 0,	0 6 0,	1 2 0,	1 3 0,	1 4 0,
1 5 0,	1 6 0,	1 7 0,	2 3 0,	2 4 0,	2 5 0,
2 6 0,	2 7 0,	3 3 0,	3 4 0,	3 5 0,	3 6 0,
3 7 0,	4 4 0,	4 5 0,	4 6 0,	4 7 0,	5 5 0,
5 6 0,	0 2 1,	0 3 1,	0 4 1,	0 5 1,	0 6 1,
0 7 1,	1 2 1,	1 3 1,	1 4 1,	1 5 1,	1 6 1,
1 7 1,	2 3 1,	2 4 1,	2 5 1,	2 6 1,	2 7 1,
3 4 1,	3 5 1,	3 6 1,	3 7 1,	4 4 1,	4 5 1,
4 6 1,	4 7 1,	5 5 1,	5 6 1,	0 2 2,	0 3 2,
0 4 2,	0 5 2,	0 6 2,	0 7 2,	1 2 2,	1 3 2,
1 4 2,	1 5 2,	1 6 2,	1 7 2,	2 3 2,	2 4 2,
2 5 2,	2 6 2,	2 7 2,	3 4 2,	3 5 2,	3 6 2,
3 7 2,	4 4 2,	4 5 2,	4 6 2,	4 7 2,	5 5 2,
5 6 2,	0 1 3,	0 2 3,	0 3 3,	0 4 3,	0 5 3,
0 6 3,	0 7 3,	1 2 3,	1 3 3,	1 4 3,	1 5 3,
1 6 3,	1 7 3,	2 3 3,	2 4 3,	2 5 3,	2 6 3,

PLAT915_ALERT_3_B No Flack x Check Done: Low Friedel Pair Coverage 27 %

Alert level C

PLAT089_ALERT_3_C Poor Data / Parameter Ratio (Zmax < 18) .... 6.71 Note
PLAT234_ALERT_4_C Large Hirshfeld Difference S1 --C11 . 0.17 Ang.

Alert level G

ABSMU01_ALERT_1_G Calculation of _exptl_absorpt_correction_mu
not performed for this radiation type.
PLAT032_ALERT_4_G Std. Uncertainty on Flack Parameter Value High . 0.300 Report
PLAT072_ALERT_2_G SHELXL First Parameter in WGHT Unusually Large 0.20 Report
PLAT092_ALERT_4_G Check: Wavelength Given is not Cu,Ga,Mo,Ag,In Ka 0.49225 Ang.
PLAT199_ALERT_1_G Reported _cell_measurement_temperature ..... (K) 293 Check
PLAT200_ALERT_1_G Reported _diffrn_ambient_temperature ..... (K) 293 Check

```
PLAT432_ALERT_2_G Short Inter X...Y Contact  S1        ..C11      .       3.28 Ang.
                                            -x,-1/2+y,3/2-z  =      4_546 Check
PLAT432_ALERT_2_G Short Inter X...Y Contact  O2        ..C4       .       2.99 Ang.
                                                   -1+x,y,z  =      1_455 Check
PLAT432_ALERT_2_G Short Inter X...Y Contact  C4        ..C9       .       3.18 Ang.
                                                    1+x,y,z  =      1_655 Check
PLAT910_ALERT_3_G Missing # of FCF Reflection(s) Below Theta(Min).          3 Note
                0  1  1,   0  0  2,   0  1  2,
PLAT912_ALERT_4_G Missing # of FCF Reflections Above STh/L=  0.600        349 Note
PLAT913_ALERT_3_G Missing # of Very Strong Reflections in FCF ....          1 Note
                0  2  2,
PLAT941_ALERT_3_G Average HKL Measurement Multiplicity ..........         4.7 Low
PLAT951_ALERT_5_G Calculated (ThMax) and CIF-Reported Kmax Differ           6 Units
PLAT952_ALERT_5_G Calculated (ThMax) and CIF-Reported Lmax Differ.          4 Units
PLAT957_ALERT_1_G Calculated (ThMax) and Actual (FCF) Kmax Differ           6 Units
PLAT958_ALERT_1_G Calculated (ThMax) and Actual (FCF) Lmax Differ.          4 Units
PLAT978_ALERT_2_G Number C-C Bonds with Positive Residual Density.          3 Info
PLAT984_ALERT_1_G The  C-f'=   -0.0004 Deviates from the B&C-Value     0.0006 Check
PLAT984_ALERT_1_G The  O-f'=    0.0008 Deviates from the B&C-Value     0.0038 Check
PLAT984_ALERT_1_G The  S-f'=    0.0506 Deviates from the B&C-Value     0.0646 Check
```

---

1 **ALERT level A** = Most likely a serious problem - resolve or explain
3 **ALERT level B** = A potentially serious problem, consider carefully
2 **ALERT level C** = Check. Ensure it is not caused by an omission or oversight
21 **ALERT level G** = General information/check it is not something unexpected

8 ALERT type 1 CIF construction/syntax error, inconsistent or missing data
5 ALERT type 2 Indicator that the structure model may be wrong or deficient
8 ALERT type 3 Indicator that the structure quality may be low
4 ALERT type 4 Improvement, methodology, query or suggestion
2 ALERT type 5 Informative message, check

Datablock: Ylid_3p23

Bond precision:	C-C = 0.0135 A		Wavelength=0.49255
Cell:	a=5.6203(3)	b=8.346(2)	c=17.5686(18)
	alpha=90	beta=90	gamma=90
Temperature:	293 K		

	Calculated	Reported
Volume	824.1(2)	824.1(2)
Space group	P 21 21 21	P 21 21 21
Hall group	P 2ac 2ab	P 2ac 2ab
Moiety formula	C11 H10 O2 S	C11 H10 O2 S
Sum formula	C11 H10 O2 S	C11 H10 O2 S
Mr	206.25	206.25
Dx,g cm-3	1.662	1.662
Z	4	4
Mu (mm-1)	0.138	0.138
F000	432.0	432.0
F000'	432.27	
h,k,lmax	9,13,28	8,7,24
Nref	3746[2165]	1248
Tmin,Tmax	0.987,0.993	0.167,1.000
Tmin'	0.984	

Correction method= # Reported T Limits: Tmin=0.167 Tmax=1.000
AbsCorr = MULTI-SCAN

Data completeness= 0.58/0.33 Theta(max)= 23.687

R(reflections)= 0.0984(1038) wR2(reflections)= 0.2571(1248)

S = 1.134 Npar= 129

The following ALERTS were generated. Each ALERT has the format
test-name_ALERT_alert-type_alert-level.
Click on the hyperlinks for more details of the test.

Alert level A

PLAT029_ALERT_3_A _diffrn_measured_fraction_theta_full value Low . 0.566 Why?

Author Response: Our experiment was done using a diamond-anvil cell rotated about one axis by 76 deg. Hence, due to the strategy and a limited access to the sample in a high-pressure cell no complete data was collected.

Alert level B

PLAT340_ALERT_3_B Low Bond Precision on C-C Bonds .... 0.0135 Ang.
PLAT911_ALERT_3_B Missing FCF Refl Between Thmin & STh/L= 0.600 309 Report

```
0 2 0,   0 4 0,   0 6 0,   1 2 0,   1 3 0,   1 4 0,
1 5 0,   1 6 0,   1 7 0,   2 3 0,   2 4 0,   2 5 0,
2 6 0,   2 7 0,   3 3 0,   3 4 0,   3 5 0,   3 6 0,
3 7 0,   4 4 0,   4 5 0,   4 6 0,   4 7 0,   5 4 0,
```

```
                5  5  0,    5  6  0,    0  2  1,    0  3  1,    0  4  1,    0  5  1,
                0  6  1,    0  7  1,    1  2  1,    1  3  1,    1  4  1,    1  5  1,
                1  6  1,    1  7  1,    2  3  1,    2  4  1,    2  5  1,    2  6  1,
                2  7  1,    3  4  1,    3  5  1,    3  6  1,    3  7  1,    4  4  1,
                4  5  1,    4  6  1,    4  7  1,    5  5  1,    5  6  1,    0  2  2,
                0  3  2,    0  4  2,    0  5  2,    0  6  2,    0  7  2,    1  2  2,
                1  3  2,    1  4  2,    1  5  2,    1  6  2,    1  7  2,    2  3  2,
                2  4  2,    2  5  2,    2  6  2,    2  7  2,    3  4  2,    3  5  2,
                3  6  2,    3  7  2,    4  4  2,    4  5  2,    4  6  2,    4  7  2,
                5  5  2,    5  6  2,    0  1  3,    0  2  3,    0  3  3,    0  4  3,
                0  5  3,    0  6  3,    0  7  3,    1  3  3,    1  4  3,    1  5  3,
                1  6  3,    1  7  3,    2  3  3,    2  4  3,    2  5  3,    2  6  3,
PLAT915_ALERT_3_B No Flack x Check Done: Low Friedel Pair Coverage         25 %
```

Alert level C

```
STRVA01_ALERT_4_C           Flack parameter is too small
           From the CIF: _refine_ls_abs_structure_Flack   -0.500
           From the CIF: _refine_ls_abs_structure_Flack_su    0.300
PLAT084_ALERT_3_C High wR2 Value (i.e. > 0.25) ...................       0.26 Report
PLAT089_ALERT_3_C Poor Data / Parameter Ratio (Zmax < 18) ........       6.64 Note
PLAT234_ALERT_4_C Large Hirshfeld Difference S1       --C11      .       0.19 Ang.
PLAT975_ALERT_2_C Check Calcd Resid. Dens.  0.87Ang From O2      .       0.40 eA-3
PLAT975_ALERT_2_C Check Calcd Resid. Dens.  1.05Ang From O1      .       0.40 eA-3
```

Alert level G

```
ABSMU01_ALERT_1_G  Calculation of _exptl_absorpt_correction_mu
                not performed for this radiation type.
PLAT032_ALERT_4_G Std. Uncertainty on Flack Parameter Value High .      0.300 Report
PLAT072_ALERT_2_G SHELXL First  Parameter in WGHT  Unusually Large       0.20 Report
PLAT092_ALERT_4_G Check: Wavelength Given is not Cu,Ga,Mo,Ag,In Ka    0.49255 Ang.
PLAT199_ALERT_1_G Reported _cell_measurement_temperature ..... (K)        293 Check
PLAT200_ALERT_1_G Reported   _diffrn_ambient_temperature ..... (K)        293 Check
PLAT432_ALERT_2_G Short Inter X...Y Contact  S1       ..C11      .       3.28 Ang.
                                         -x,-1/2+y,3/2-z  =      4_546 Check
PLAT432_ALERT_2_G Short Inter X...Y Contact  O2       ..C4       .       2.96 Ang.
                                                -1+x,y,z  =      1_455 Check
PLAT432_ALERT_2_G Short Inter X...Y Contact  C4       ..C9       .       3.16 Ang.
                                                 1+x,y,z  =      1_655 Check
PLAT910_ALERT_3_G Missing # of FCF Reflection(s) Below Theta(Min).          3 Note
                0  1  1,    0  0  2,    0  1  2,
PLAT912_ALERT_4_G Missing # of FCF Reflections Above STh/L=  0.600        362 Note
PLAT913_ALERT_3_G Missing # of Very Strong Reflections in FCF ....          1 Note
                0  2  2,
PLAT930_ALERT_2_G FCF-based   Twin Law         [ 0 5 1] Est.d BASF       0.60 Check
PLAT931_ALERT_5_G CIFcalcFCF  Twin Law         [ 0 5 1] Est.d BASF       0.60 Check
PLAT941_ALERT_3_G Average HKL Measurement Multiplicity ...........        4.8 Low
PLAT951_ALERT_5_G Calculated (ThMax) and CIF-Reported Kmax Differ           6 Units
PLAT952_ALERT_5_G Calculated (ThMax) and CIF-Reported Lmax Differ.          4 Units
PLAT957_ALERT_1_G Calculated (ThMax) and Actual (FCF) Kmax Differ           6 Units
PLAT958_ALERT_1_G Calculated (ThMax) and Actual (FCF) Lmax Differ.          4 Units
PLAT978_ALERT_2_G Number C-C Bonds with Positive Residual Density.          2 Info
PLAT984_ALERT_1_G The  C-f'=   -0.0004 Deviates from the B&C-Value     0.0006 Check
PLAT984_ALERT_1_G The  O-f'=    0.0008 Deviates from the B&C-Value     0.0038 Check
PLAT984_ALERT_1_G The  S-f'=    0.0507 Deviates from the B&C-Value     0.0647 Check
```

```
   1 ALERT level A = Most likely a serious problem - resolve or explain
   3 ALERT level B = A potentially serious problem, consider carefully
   6 ALERT level C = Check. Ensure it is not caused by an omission or oversight
  23 ALERT level G = General information/check it is not something unexpected

   8 ALERT type 1 CIF construction/syntax error, inconsistent or missing data
   8 ALERT type 2 Indicator that the structure model may be wrong or deficient
   9 ALERT type 3 Indicator that the structure quality may be low
   5 ALERT type 4 Improvement, methodology, query or suggestion
   3 ALERT type 5 Informative message, check
```

Datablock: Ylid_3p89

Bond precision: C-C = 0.0151 A Wavelength=0.49225

Cell:	a=5.6121(5)	b=8.225(4)	c=17.428(3)
	alpha=90	beta=90	gamma=90
Temperature:	293 K		

	Calculated	Reported
Volume	804.5(4)	804.4(4)
Space group	P 21 21 21	P 21 21 21
Hall group	P 2ac 2ab	P 2ac 2ab
Moiety formula	C11 H10 O2 S	C11 H10 O2 S
Sum formula	C11 H10 O2 S	C11 H10 O2 S
Mr	206.25	206.25
Dx,g cm-3	1.703	1.703
Z	4	4
Mu (mm-1)	0.141	0.141
F000	432.0	432.0
F000’	432.27	
h,k,lmax	9,13,28	8,6,24
Nref	3679[2129]	1186
Tmin,Tmax	0.987,0.993	0.346,1.000
Tmin’	0.983	

Correction method= # Reported T Limits: Tmin=0.346 Tmax=1.000
AbsCorr = MULTI-SCAN

Data completeness= 0.56/0.32 Theta(max)= 23.706

R(reflections)= 0.1097(911) wR2(reflections)= 0.2861(1186)

S = 1.186 Npar= 99

The following ALERTS were generated. Each ALERT has the format
test-name_ALERT_alert-type_alert-level.
Click on the hyperlinks for more details of the test.

Alert level A

```
PLAT029_ALERT_3_A _diffrn_measured_fraction_theta_full value Low .      0.547 Why?
```

Author Response: Our experiment was done using a diamond-anvil cell rotated about one axis by 76 deg. Hence, due to the strategy and a limited access to the sample in a high-pressure cell no complete data was collected.

Alert level B

```
PLAT340_ALERT_3_B Low Bond Precision on  C-C Bonds ...............     0.0151 Ang.
PLAT911_ALERT_3_B Missing FCF Refl Between Thmin & STh/L=    0.600        262 Report
                0  2  0,   0  4  0,   0  6  0,   1  2  0,   1  3  0,   1  4  0,
                1  5  0,   1  6  0,   2  0  0,   2  3  0,   2  4  0,   2  5  0,
                2  6  0,   3  3  0,   3  4  0,   3  5  0,   3  6  0,   4  4  0,
                4  5  0,   4  6  0,   5  4  0,   5  5  0,   5  6  0,   6  0  0,
                0  2  1,   0  3  1,   0  4  1,   0  5  1,   0  6  1,   1  2  1,
                1  3  1,   1  4  1,   1  5  1,   1  6  1,   2  0  1,   2  3  1,
                2  4  1,   2  5  1,   2  6  1,   3  3  1,   3  4  1,   3  5  1,
                3  6  1,   4  4  1,   4  5  1,   4  6  1,   5  5  1,   5  6  1,
                0  2  2,   0  3  2,   0  4  2,   0  5  2,   0  6  2,   1  2  2,
                1  3  2,   1  4  2,   1  5  2,   1  6  2,   2  3  2,   2  4  2,
                2  5  2,   2  6  2,   3  4  2,   3  5  2,   3  6  2,   4  4  2,
                4  5  2,   4  6  2,   5  5  2,   5  6  2,   0  2  3,   0  3  3,
                0  4  3,   0  5  3,   0  6  3,   1  2  3,   1  3  3,   1  4  3,
                1  5  3,   1  6  3,   2  3  3,   2  4  3,   2  5  3,   2  6  3,
                3  4  3,   3  5  3,   3  6  3,   4  4  3,   4  5  3,   4  6  3,
                5  5  3,   5  6  3,   0  0  4,   0  1  4,   0  2  4,   0  3  4,
PLAT915_ALERT_3_B No Flack x Check Done: Low Friedel Pair Coverage         24 %
```

Alert level C

```
STRVA01_ALERT_4_C           Flack parameter is too small
           From the CIF: _refine_ls_abs_structure_Flack   -0.400
           From the CIF: _refine_ls_abs_structure_Flack_su    0.400
PLAT082_ALERT_2_C High R1 Value .................................       0.11 Report
PLAT084_ALERT_3_C High wR2 Value (i.e. > 0.25) ...................       0.29 Report
PLAT094_ALERT_2_C Ratio of Maximum / Minimum Residual Density ....       2.04 Report
PLAT910_ALERT_3_C Missing # of FCF Reflection(s) Below Theta(Min).          5 Note
                0  1  1,   1  0  1,   0  0  2,   0  1  2,   0  1  3,
```

Alert level G

```
ABSMU01_ALERT_1_G  Calculation of _exptl_absorpt_correction_mu
                not performed for this radiation type.
PLAT032_ALERT_4_G Std. Uncertainty on Flack Parameter Value High .      0.400 Report
PLAT072_ALERT_2_G SHELXL First  Parameter in WGHT  Unusually Large       0.20 Report
PLAT092_ALERT_4_G Check: Wavelength Given is not Cu,Ga,Mo,Ag,In Ka    0.49225 Ang.
```

```
PLAT171_ALERT_4_G The CIF-Embedded .res File Contains EADP Records          1 Report
PLAT199_ALERT_1_G Reported _cell_measurement_temperature ..... (K)        293 Check
PLAT200_ALERT_1_G Reported   _diffrn_ambient_temperature ..... (K)        293 Check
PLAT432_ALERT_2_G Short Inter X...Y Contact  S1       ..C11      .       3.26 Ang.
                                          -x,-1/2+y,3/2-z  =      4_546 Check
PLAT432_ALERT_2_G Short Inter X...Y Contact  O2       ..C4       .       2.91 Ang.
                                                 -1+x,y,z  =      1_455 Check
PLAT432_ALERT_2_G Short Inter X...Y Contact  C2       ..C5       .       3.18 Ang.
                                         -1/2+x,3/2-y,1-z  =      3_466 Check
PLAT432_ALERT_2_G Short Inter X...Y Contact  C4       ..C9       .       3.10 Ang.
                                                  1+x,y,z  =      1_655 Check
PLAT912_ALERT_4_G Missing # of FCF Reflections Above STh/L=  0.600        281 Note
PLAT913_ALERT_3_G Missing # of Very Strong Reflections in FCF ....          1 Note
                0  2  2,
PLAT933_ALERT_2_G Number of HKL-OMIT Records in Embedded .res File          4 Note
                2  0  0,   2  0  1,   5  3 15,   1  0  1,
PLAT951_ALERT_5_G Calculated (ThMax) and CIF-Reported Kmax Differ           7 Units
PLAT952_ALERT_5_G Calculated (ThMax) and CIF-Reported Lmax Differ.          4 Units
PLAT957_ALERT_1_G Calculated (ThMax) and Actual (FCF) Kmax Differ           7 Units
PLAT958_ALERT_1_G Calculated (ThMax) and Actual (FCF) Lmax Differ.          4 Units
PLAT978_ALERT_2_G Number C-C Bonds with Positive Residual Density.          6 Info
PLAT984_ALERT_1_G The  C-f'=   -0.0004 Deviates from the B&C-Value     0.0006 Check
PLAT984_ALERT_1_G The  O-f'=    0.0008 Deviates from the B&C-Value     0.0038 Check
PLAT984_ALERT_1_G The  S-f'=    0.0506 Deviates from the B&C-Value     0.0646 Check
```

---

1 **ALERT level A** = Most likely a serious problem - resolve or explain
3 **ALERT level B** = A potentially serious problem, consider carefully
5 **ALERT level C** = Check. Ensure it is not caused by an omission or oversight
22 **ALERT level G** = General information/check it is not something unexpected

8 ALERT type 1 CIF construction/syntax error, inconsistent or missing data
9 ALERT type 2 Indicator that the structure model may be wrong or deficient
7 ALERT type 3 Indicator that the structure quality may be low
5 ALERT type 4 Improvement, methodology, query or suggestion
2 ALERT type 5 Informative message, check

It is advisable to attempt to resolve as many as possible of the alerts in all categories. Often the minor alerts point to easily fixed oversights, errors and omissions in your CIF or refinement strategy, so attention to these fine details can be worthwhile. In order to resolve some of the more serious problems it may be necessary to carry out additional measurements or structure refinements. However, the purpose of your study may justify the reported deviations and the more serious of these should normally be commented upon in the discussion or experimental section of a paper or in the "special_details" fields of the CIF. checkCIF was carefully designed to identify outliers and unusual parameters, but every test has its limitations and alerts that are not important in a particular case may appear. Conversely, the absence of alerts does not guarantee there are no aspects of the results needing attention. It is up to the individual to critically assess their own results and, if necessary, seek expert advice.

Publication of your CIF in IUCr journals

A basic structural check has been run on your CIF. These basic checks will be run on all CIFs submitted for publication in IUCr journals (*Acta Crystallographica*, *Journal of Applied Crystallography*, *Journal of Synchrotron Radiation*); however, if you intend to submit to *Acta Crystallographica Section C* or *E* or *IUCrData*, you should make sure that full publication checks are run on the final version of your CIF prior to submission.

Publication of your CIF in other journals

Please refer to the *Notes for Authors* of the relevant journal for any special instructions relating to CIF submission.

PLATON version of 29/11/2023; check.def file version of 14/09/2023

Datablock Ylid_0p00001 - ellipsoid plot

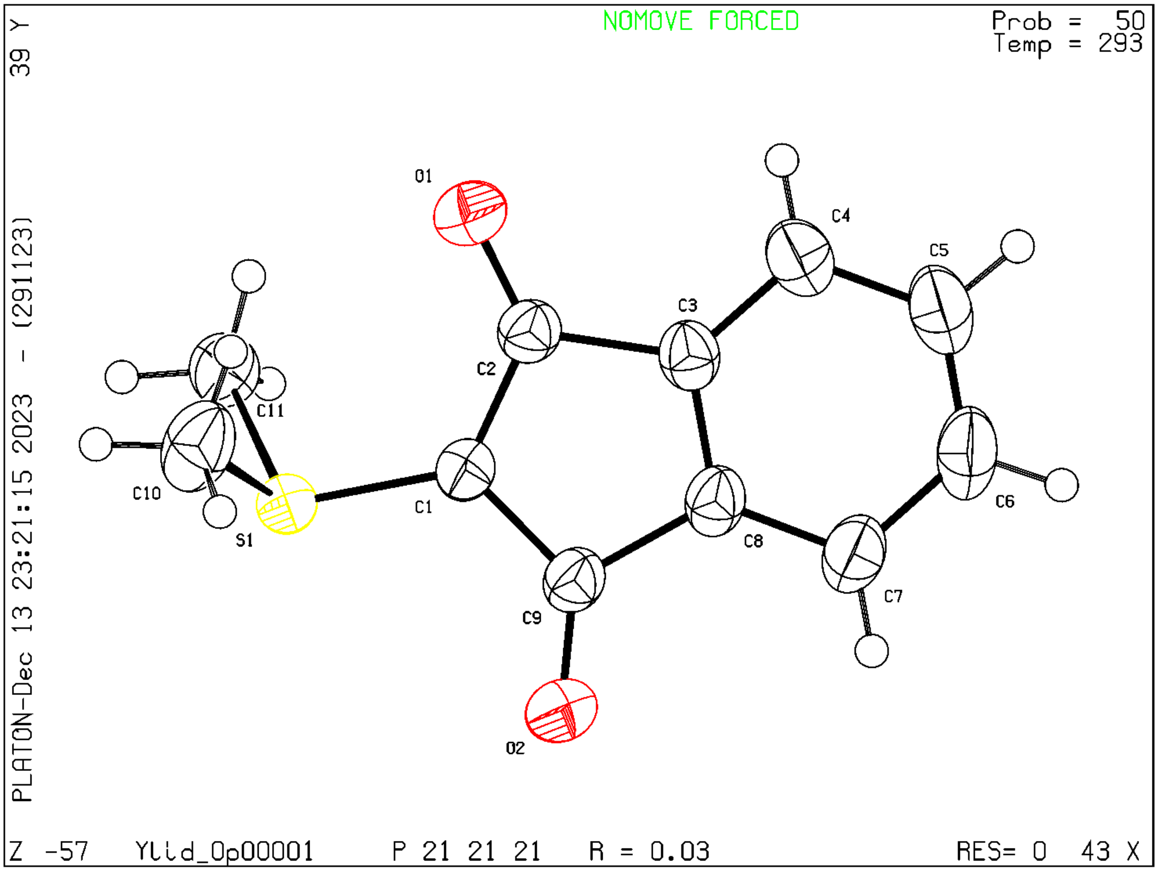

Datablock Ylid_0p12 - ellipsoid plot

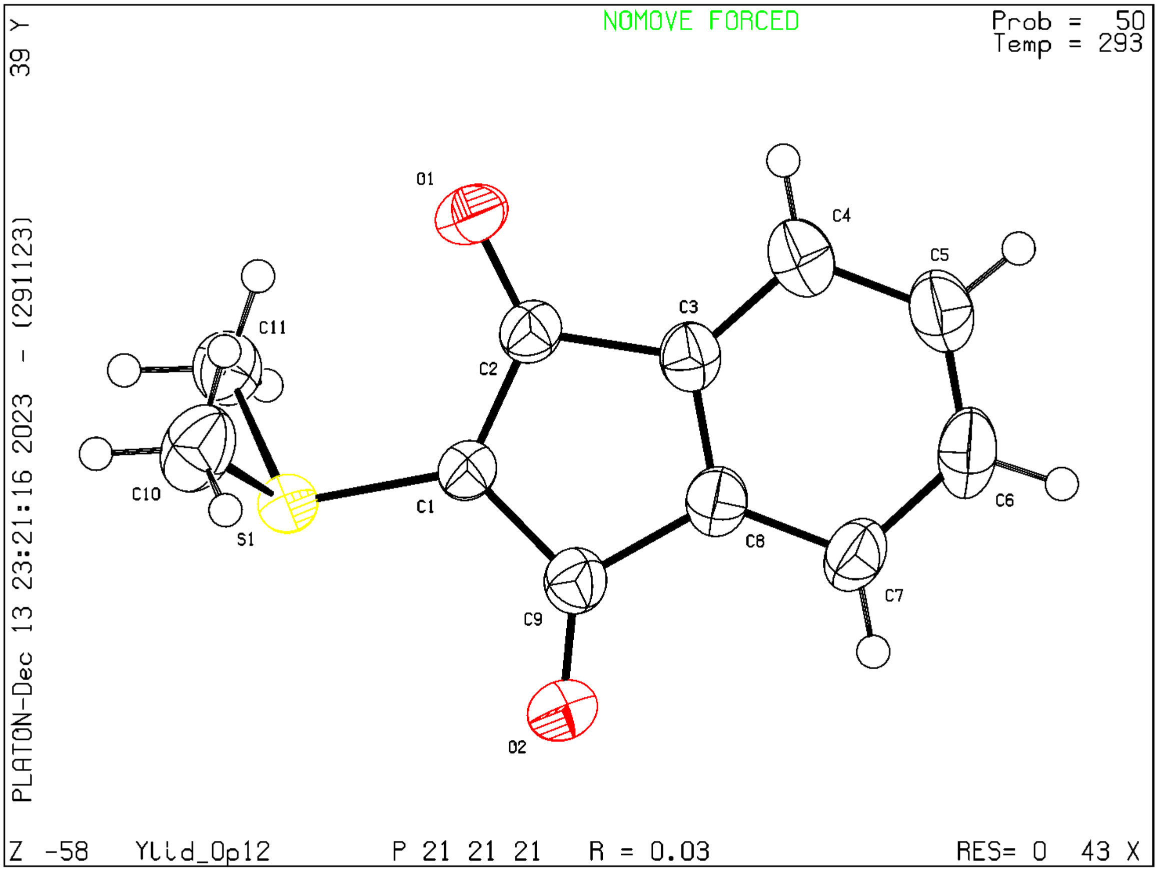

Datablock Ylid_0p59 - ellipsoid plot

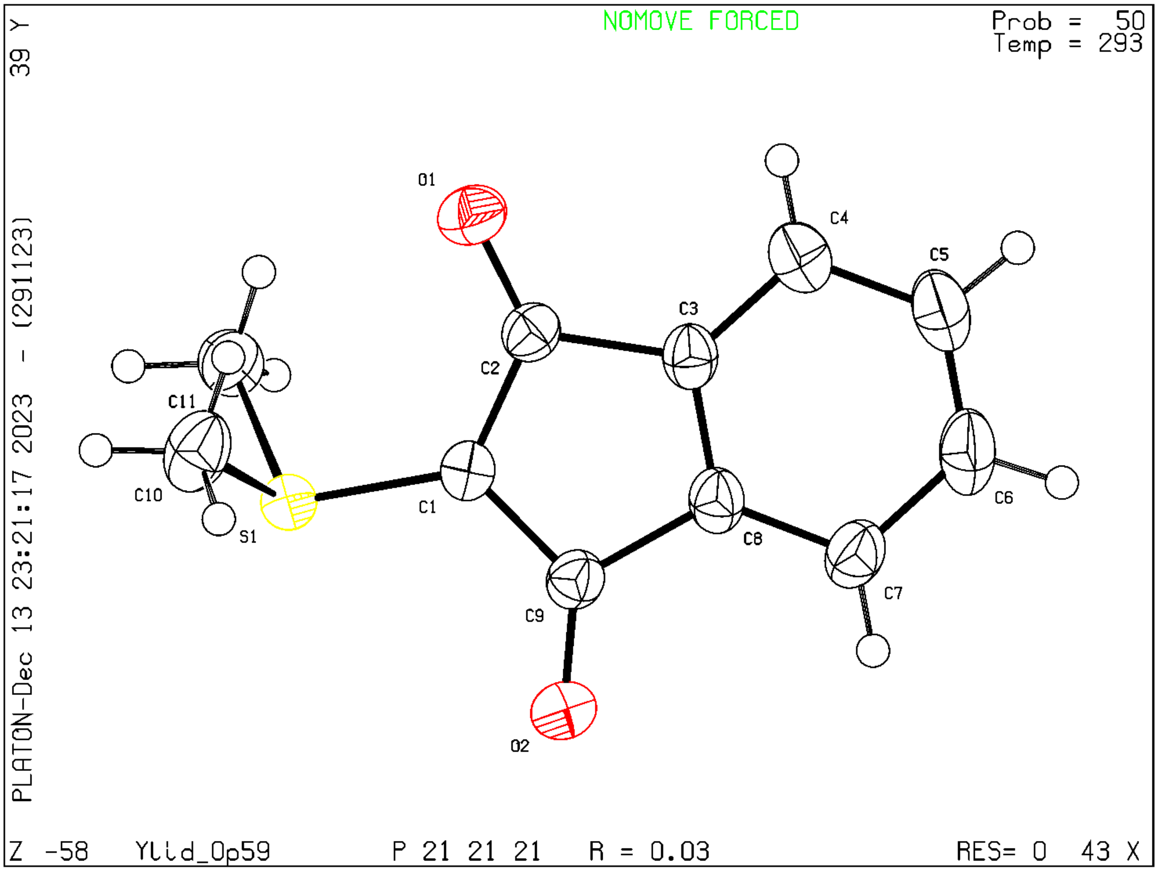

Datablock Ylid_2p12 - ellipsoid plot

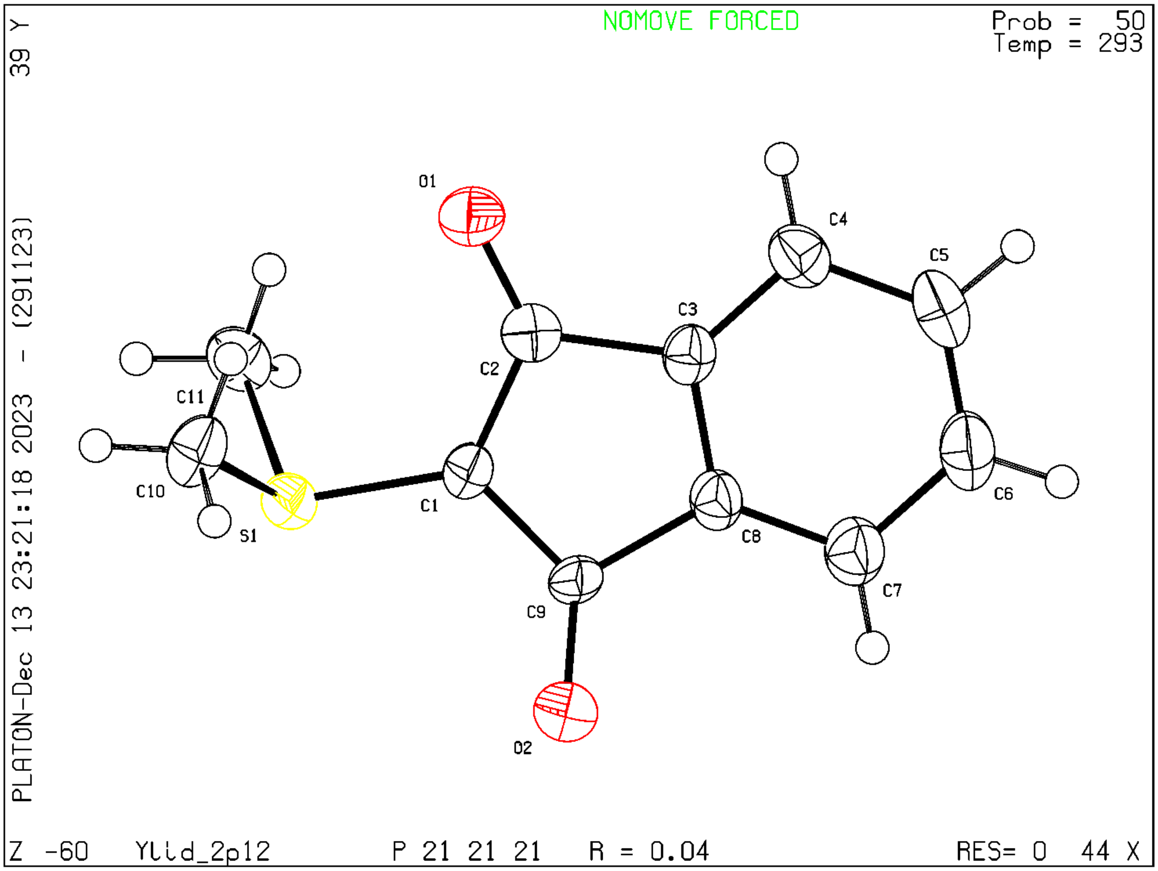

Datablock Ylid_2p82 - ellipsoid plot

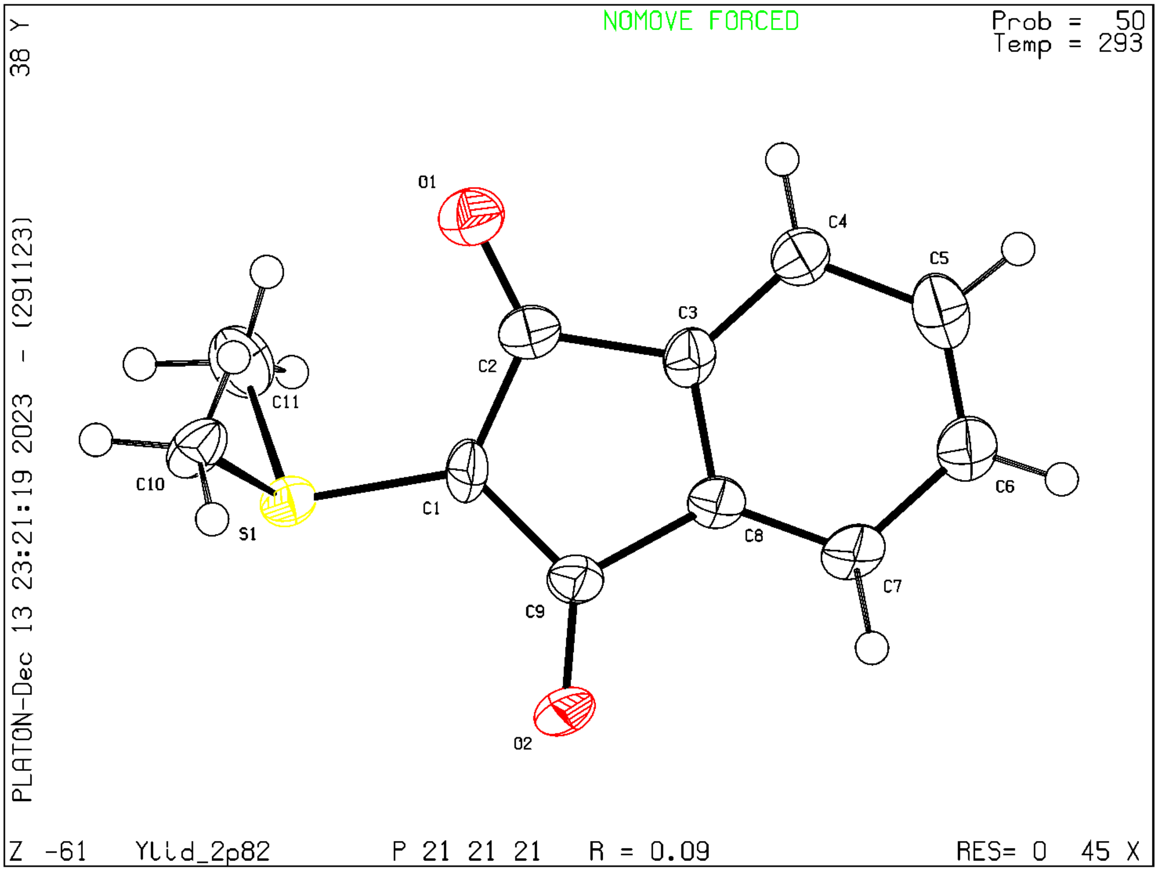

Datablock Ylid_3p23 - ellipsoid plot

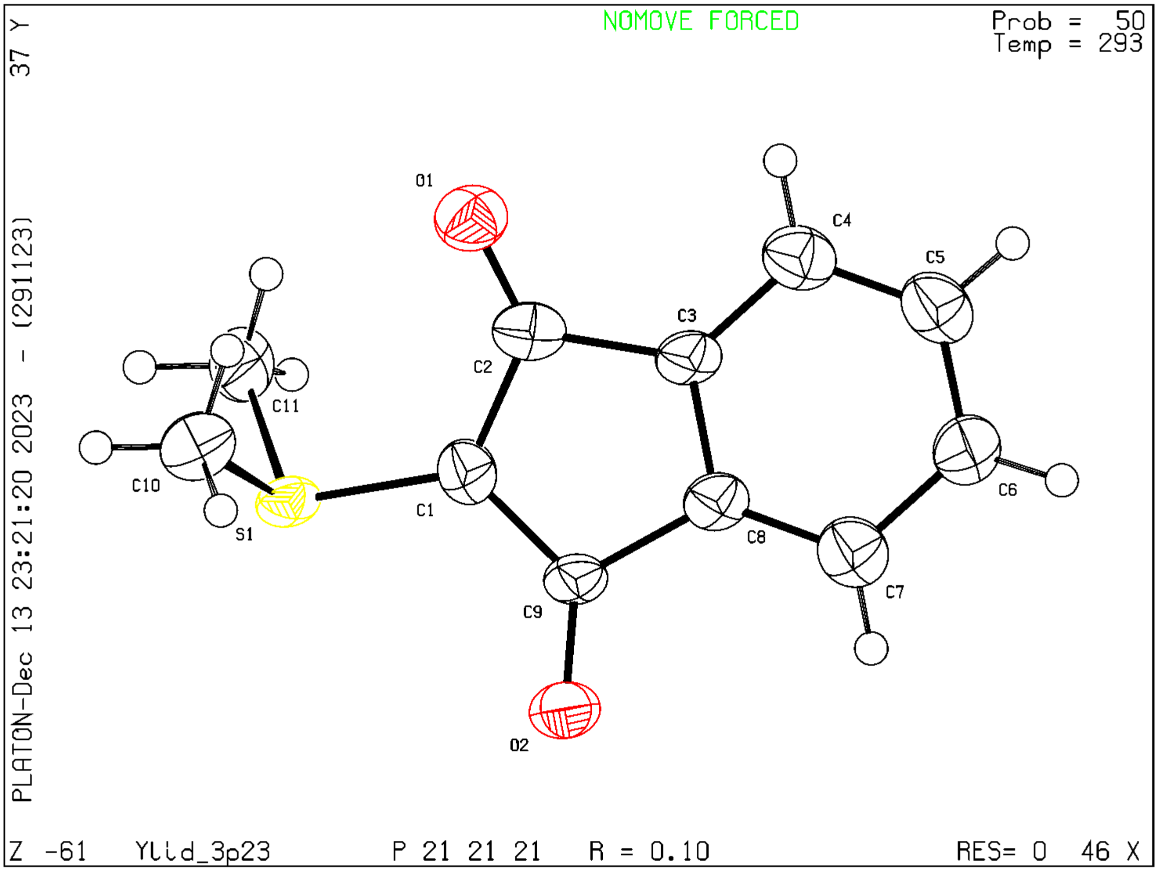

Datablock Ylid_3p89 - ellipsoid plot

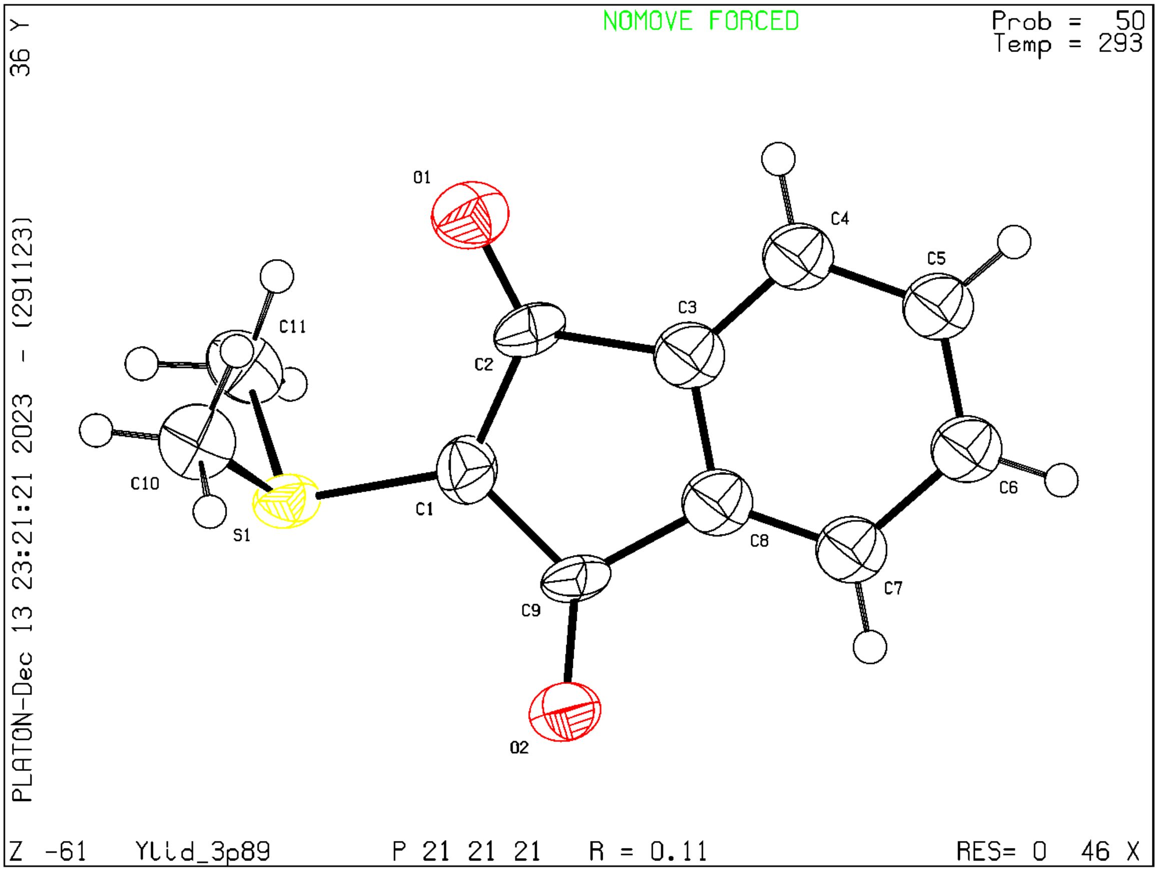

Supplement: Supplementary file 3 — Supplementary Information 3. [file 41598_2025_95269_MOESM3_ESM.zip › Ylid_HP.pdf]
