# Supplementary material for: Application of a quantum crystallographic protocol to YLID, the world's most common crystal structure
Source: Sci Rep. 2025 Apr 29;15:15045. doi: 10.1038/s41598-025-95269-3 (PMC12041244; doi:10.1038/s41598-025-95269-3)

checkCIF/PLATON report

Structure factors have been supplied for datablock(s) Ylide_2H2O_twin_cut_G1_twin1_hklf4

THIS REPORT IS FOR GUIDANCE ONLY. IF USED AS PART OF A REVIEW PROCEDURE FOR PUBLICATION, IT SHOULD NOT REPLACE THE EXPERTISE OF AN EXPERIENCED CRYSTALLOGRAPHIC REFEREE.

No syntax errors found.   CIF dictionary   Interpreting this report

Datablock: Ylide_2H2O_twin_cut_G1_twin1_hklf4

Bond precision:	C-C = 0.0005 A		Wavelength=0.71073
Cell:	a=6.9985(1)	b=9.3869(1)	c=32.0797(2)
	alpha=90	beta=92.044(1)	gamma=90
Temperature:	100 K		

	Calculated	Reported
Volume	2106.11(4)	2106.11(4)
Space group	P 21/c	P 1 21/c 1
Hall group	-P 2ybc	-P 2ybc
Moiety formula	C11 H10 O2 S, H2 O	C11 H10 O2 S, H2 O
Sum formula	C11 H12 O3 S	C11 H12 O3 S
Mr	224.27	224.28
Dx,g cm-3	1.415	1.415
Z	8	8
Mu (mm-1)	0.290	0.290
F000	944.0	945.5
F000'	945.45	
h,k,lmax	12,16,55	12,16,55
Nref	11639	23240
Tmin,Tmax	0.962,0.983	0.967,0.986
Tmin'	0.949	

Correction method= # Reported T Limits: Tmin=0.967 Tmax=0.986
AbsCorr = GAUSSIAN

Data completeness= 1.997   Theta(max)= 38.210

R(reflections)= 0.0261(16688)   wR2(reflections)= 0.0701(23240)

S = 1.029   Npar= 488

The following ALERTS were generated. Each ALERT has the format
test-name_ALERT_alert-type_alert-level.
Click on the hyperlinks for more details of the test.

Alert level A

PLAT992_ALERT_5_A Repd & Actual _reflns_number_gt Values Differ by 7497 Check

Author Response: This keyword calculates values with respect to F>4sigma(F). However, the refinement in Tonto does not use any cutoff and consequently produces all statistics based on F>0sigma(F). In the Tonto CIF, _reflns_number_gt and reflns_number_total and _refine_ls_number_reflns are always the same number. We do not alter these numbers just to pass the checkCIF routine.

Alert level B

PLAT921_ALERT_1_B R1 in the CIF and FCF Differ by .... 0.0105 Check

Author Response: This dataset is twinn. Shelx software can refine the structure using a twin law. However, to use NospherA2 and Olex2.refine, we had to detwinned the reflection files for use in NospherA2 to perform HAR and the inconsistency with fcf arises from here.

PLAT922_ALERT_1_B wR2 in the CIF and FCF Differ by .... 0.0244 Check

Author Response: This dataset is twinn. Shelx software can refine the structure using a twin law. However, to use NospherA2 and Olex2.refine, we had to detwinned the reflection files for use in NospherA2 to perform HAR and the inconsistency with fcf arises from here.

Alert level C

```
PLAT230_ALERT_2_C Hirshfeld Test Diff for    S1       --C21       .        6.9 s.u.
PLAT230_ALERT_2_C Hirshfeld Test Diff for    S1       --C22       .        5.9 s.u.
PLAT230_ALERT_2_C Hirshfeld Test Diff for    S2       --C10       .        5.9 s.u.
PLAT230_ALERT_2_C Hirshfeld Test Diff for    S2       --C11       .        6.4 s.u.
PLAT415_ALERT_2_C Short Inter D-H..H-X       H4A      ..H11B      .       2.15 Ang.
                                               1-x,1-y,1-z  =      3_666 Check
PLAT910_ALERT_3_C Missing # of FCF Reflection(s) Below Theta(Min).           8 Note
                1  0  0,   0  1  1,  -1  0  2,   0  0  2,   1  0  2,   0  1  2,
                0  1  3,   0  0  4,
PLAT911_ALERT_3_C Missing FCF Refl Between Thmin & STh/L=    0.600          36 Report
                3  1  0,  -3  1  1,  -3  1  2,   1  1  2,   3  1  2,  -7  2  2,
                0  2  2,   1  5  2,  -1  1  3,   1  1  3,  -7  2  3,  -1  2  3,
                0  2  3,   1  2  3,   0  3  3,  -3  6  3,  -1  1  4,   1  1  4,
```

```
                 -7  2  4,  -7  4  4,   1  7  4,   1  1  5,  -7  2  5,  -7  4  5,
                  3  6  5,   1  7  5,  -7  4  6,   5  4  6,  -2  6  6,   2  6  6,
                  1  7  6,  -7  4  7,   1  7  7,   1  6  8,   1  2 13,   2  7 13,
PLAT923_ALERT_1_C S    Values in the CIF and FCF Differ by .......      0.375 Check
```

Author Response: This dataset is twinn. Shelx software can refine the structure using a twin law. However, to use NospherA2 and Olex2.refine, we had to detwinned the reflection files for use in NospherA2 to perform HAR and the inconsistency with fcf arises from here.

Alert level G

```
PLAT003_ALERT_2_G Number of Uiso or U(i,j)  Restrained non-H-Atoms          6 Report
PLAT066_ALERT_1_G Predicted and Reported Tmin&Tmax Range Identical          ? Check
PLAT143_ALERT_4_G s.u. on c - Axis Small or Missing .............    0.00020 Ang.
PLAT187_ALERT_4_G The CIF-Embedded .res File Contains RIGU Records          5 Report
PLAT190_ALERT_3_G A Non-default RIGU Restraint Value for First Par     0.0008 Report
PLAT190_ALERT_3_G A Non-default RIGU Restraint Value for SecondPar     0.0008 Report
PLAT190_ALERT_3_G A Non-default RIGU Restraint Value for First Par     0.0005 Report
PLAT190_ALERT_3_G A Non-default RIGU Restraint Value for SecondPar     0.0005 Report
PLAT190_ALERT_3_G A Non-default RIGU Restraint Value for First Par     0.0005 Report
PLAT190_ALERT_3_G A Non-default RIGU Restraint Value for SecondPar     0.0005 Report
PLAT190_ALERT_3_G A Non-default RIGU Restraint Value for First Par     0.0005 Report
PLAT190_ALERT_3_G A Non-default RIGU Restraint Value for SecondPar     0.0005 Report
PLAT190_ALERT_3_G A Non-default RIGU Restraint Value for First Par     0.0005 Report
PLAT190_ALERT_3_G A Non-default RIGU Restraint Value for SecondPar     0.0005 Report
PLAT802_ALERT_4_G CIF Input Record(s) with more than 80 Characters          1 Info
PLAT860_ALERT_3_G Number of Least-Squares Restraints ............          18 Note
PLAT870_ALERT_4_G ALERTS Related to Twinning Effects Suppressed ..          ! Info
PLAT883_ALERT_1_G Absent Datum for _atom_sites_solution_primary ..     Please Do !
PLAT912_ALERT_4_G Missing # of FCF Reflections Above STh/L=  0.600         74 Note
PLAT933_ALERT_2_G Number of HKL-OMIT Records in Embedded .res File         37 Note
                 -7  2  2,  -7  2  3,  -7  2  4,  -7  2  5,  -7  4  4,  -7  4  5,
                 -7  4  6,  -7  4  7,  -5 14  2,  -3  1  1,  -3  1  2,  -3  6  3,
                 -2  6  6,  -1  1  3,  -1  1  4,  -1  2  3,   0  2  2,   0  2  3,
                  0  3  3,   1  1  2,   1  1  3,   1  1  4,   1  1  5,   1  2  3,
                  1  2 13,   1  5  2,   1  6  8,   1  7  4,   1  7  5,   1  7  6,
                  1  7  7,   2  6  6,   2  7 13,   3  1  0,   3  1  2,   3  6  5,
                  5  4  6,
PLAT962_ALERT_5_G Number  FCF File  Relections with Sigma(I) = 0.0          1 Note
PLAT969_ALERT_5_G The 'Henn et al.' R-Factor-gap value ...........      4.549 Note
              Predicted wR2: Based on SigI**2  1.01 or SHELX Weight  7.00
PLAT979_ALERT_1_G NoSpherA2 Scattering Factors Used .............     Please Note
```

---

1 **ALERT level A** = Most likely a serious problem - resolve or explain
2 **ALERT level B** = A potentially serious problem, consider carefully
8 **ALERT level C** = Check. Ensure it is not caused by an omission or oversight
23 **ALERT level G** = General information/check it is not something unexpected

6 ALERT type 1 CIF construction/syntax error, inconsistent or missing data

```
 7 ALERT type 2 Indicator that the structure model may be wrong or deficient
13 ALERT type 3 Indicator that the structure quality may be low
 5 ALERT type 4 Improvement, methodology, query or suggestion
 3 ALERT type 5 Informative message, check
```

It is advisable to attempt to resolve as many as possible of the alerts in all categories. Often the minor alerts point to easily fixed oversights, errors and omissions in your CIF or refinement strategy, so attention to these fine details can be worthwhile. In order to resolve some of the more serious problems it may be necessary to carry out additional measurements or structure refinements. However, the purpose of your study may justify the reported deviations and the more serious of these should normally be commented upon in the discussion or experimental section of a paper or in the "special_details" fields of the CIF. checkCIF was carefully designed to identify outliers and unusual parameters, but every test has its limitations and alerts that are not important in a particular case may appear. Conversely, the absence of alerts does not guarantee there are no aspects of the results needing attention. It is up to the individual to critically assess their own results and, if necessary, seek expert advice.

Publication of your CIF in IUCr journals

A basic structural check has been run on your CIF. These basic checks will be run on all CIFs submitted for publication in IUCr journals (*Acta Crystallographica*, *Journal of Applied Crystallography*, *Journal of Synchrotron Radiation*); however, if you intend to submit to *Acta Crystallographica Section C* or *E* or *IUCrData*, you should make sure that full publication checks are run on the final version of your CIF prior to submission.

Publication of your CIF in other journals

Please refer to the *Notes for Authors* of the relevant journal for any special instructions relating to CIF submission.

Validation response form

Please find below a validation response form (VRF) that can be filled in and pasted into your CIF.

```
# start Validation Reply Form
_vrf_PLAT230_Ylide_2H2O_twin_cut_G1_twin1_hklf4
;
PROBLEM: Hirshfeld Test Diff for    S1       --C21      .        6.9 s.u.
RESPONSE: ...
;
_vrf_PLAT415_Ylide_2H2O_twin_cut_G1_twin1_hklf4
;
PROBLEM: Short Inter D-H..H-X       H4A      ..H11B     .       2.15 Ang.
RESPONSE: ...
;
_vrf_PLAT910_Ylide_2H2O_twin_cut_G1_twin1_hklf4
;
PROBLEM: Missing # of FCF Reflection(s) Below Theta(Min).          8 Note
RESPONSE: ...
;
```

```
_vrf_PLAT911_Ylide_2H2O_twin_cut_G1_twin1_hklf4
;
PROBLEM: Missing FCF Refl Between Thmin & STh/L=    0.600         36 Report
RESPONSE: ...
;
# end Validation Reply Form
```

PLATON version of 11/11/2024; check.def file version of 11/11/2024

Datablock Ylide_2H2O_twin_cut_G1_twin1_hklf4 - ellipsoid plot

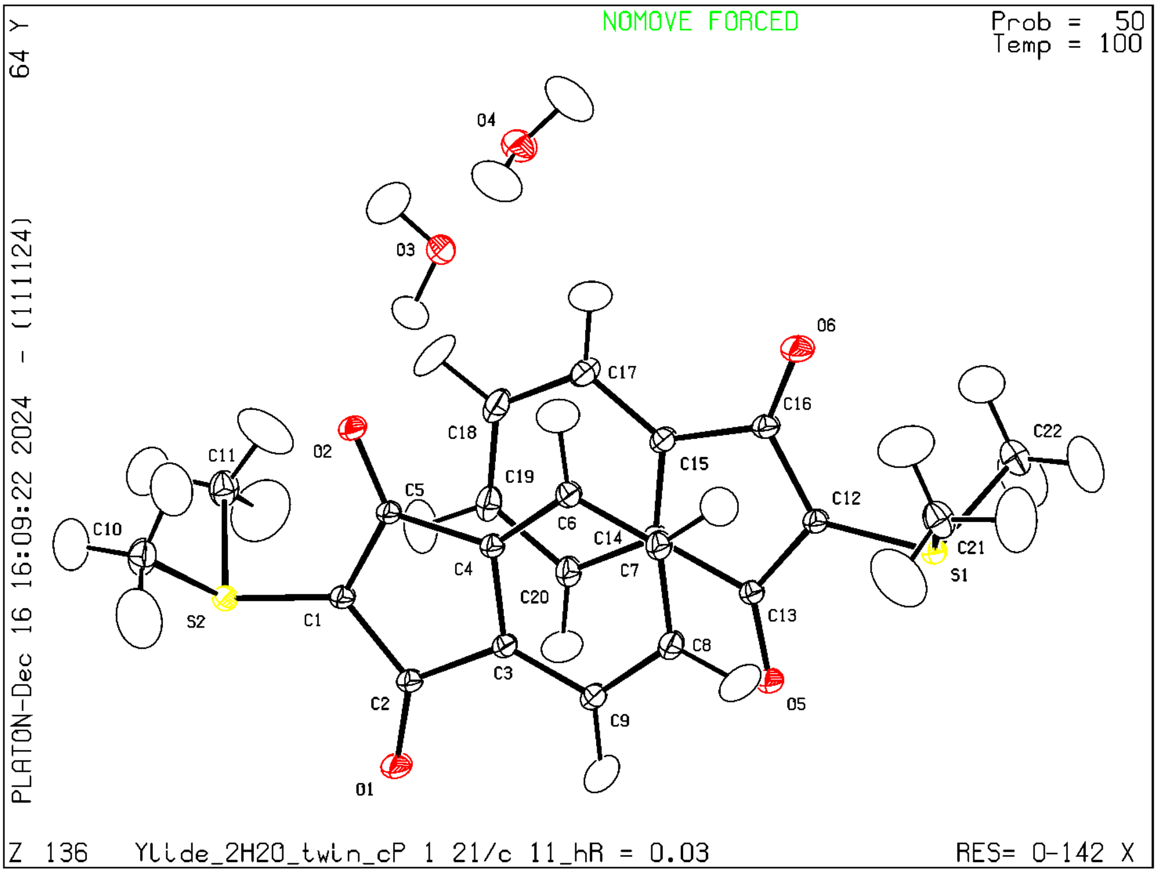

Supplement: Supplementary file 2 — Supplementary Information 2. [file Dataset_24_HAR.pdf › Dataset_24_HAR.pdf]
