# Supplementary material for: Application of a quantum crystallographic protocol to YLID, the world's most common crystal structure
Source: Sci Rep. 2025 Apr 29;15:15045. doi: 10.1038/s41598-025-95269-3 (PMC12041244; doi:10.1038/s41598-025-95269-3)

# checkCIF/PLATON report

Structure factors have been supplied for datablock(s) YLID_100K_Cu_mono

THIS REPORT IS FOR GUIDANCE ONLY. IF USED AS PART OF A REVIEW PROCEDURE FOR PUBLICATION, IT SHOULD NOT REPLACE THE EXPERTISE OF AN EXPERIENCED CRYSTALLOGRAPHIC REFEREE.

No syntax errors found.    CIF dictionary    Interpreting this report

## Datablock: YLID_100K_Cu_mono

---

| | | | |
|---|---|---|---|
| Bond precision: | C-C = 0.0010 A | | Wavelength=1.54184 |
| Cell: | a=9.45647(4) | b=10.63614(5) | c=10.49215(6) |
| | alpha=90 | beta=104.9208(5) | gamma=90 |
| Temperature: | 100 K | | |

| | Calculated | Reported |
|---|---|---|
| Volume | 1019.722(9) | 1019.722(9) |
| Space group | P 21/c | P 1 21/c 1 |
| Hall group | -P 2ybc | -P 2ybc |
| Moiety formula | C11 H10 O2 S | C11 H10 O2 S |
| Sum formula | C11 H10 O2 S | C11 H10 O2 S |
| Mr | 206.25 | 206.27 |
| Dx,g cm-3 | 1.344 | 1.344 |
| Z | 4 | 4 |
| Mu (mm-1) | 2.579 | 2.579 |
| F000 | 432.0 | 434.6 |
| F000' | 434.49 | |
| h,k,lmax | 12,13,13 | 12,13,13 |
| Nref | 2219 | 2218 |
| Tmin,Tmax | 0.447,0.582 | 0.240,1.000 |
| Tmin' | 0.383 | |

Correction method= # Reported T Limits: Tmin=0.240 Tmax=1.000
AbsCorr = MULTI-SCAN

Data completeness= 1.000    Theta(max)= 79.510

R(reflections)= 0.0151( 2218)    wR2(reflections)= 0.0345( 2218)

S = 1.047    Npar= 243

```
The following ALERTS were generated. Each ALERT has the format
       test-name_ALERT_alert-type_alert-level.
Click on the hyperlinks for more details of the test.
```

### Alert level C

```
PLAT088_ALERT_3_C Poor Data / Parameter Ratio ....................       9.13 Note
```

**Author Response: It is the purpose of this study to check if free HAR refinements of hydrogen atom positions and ADPs are reasonable for Cu data although the data to parameter ratio is naturally low because of the limited reachable resolution. Therefore, checking for overfitting is important. This is done in the following way: i) comparing the refined C-H distances with data from neutron diffraction; ii) checking the volume and direction of the H ADPs visually, and, numerically, the similarity factors of the HAR-refined H ADP values with those from SHADE 3.**

```
PLAT767_ALERT_4_C INS Embedded LIST 6 Instruction Should be LIST 4     Please Check
```

**Author Response: For further XCW fittings in Tonto, LIST 6 is necessary to include the correction of anomalous dispersion effects in the fcf file.**

### Alert level G

```
PLAT068_ALERT_1_G Reported F000 Differs from Calcd (or Missing)...     Please Check
PLAT142_ALERT_4_G s.u. on b - Axis Small or Missing .............    0.00005 Ang.
PLAT143_ALERT_4_G s.u. on c - Axis Small or Missing .............    0.00006 Ang.
PLAT802_ALERT_4_G CIF Input Record(s) with more than 80 Characters         1 Info
PLAT872_ALERT_4_G ALERTS Related to Anharmonic Refine   Suppressed         ! Info
PLAT883_ALERT_1_G Absent Datum for _atom_sites_solution_primary ..     Please Do !
PLAT912_ALERT_4_G Missing # of FCF Reflections Above STh/L=  0.600         1 Note
PLAT961_ALERT_5_G Dataset Contains no Negative Intensities ......     Please Check
PLAT969_ALERT_5_G The 'Henn et al.' R-Factor-gap value ..........      5.109 Note
              Predicted wR2: Based on SigI**2  0.68 or SHELX Weight  3.29
PLAT978_ALERT_2_G Number C-C Bonds with Positive Residual Density.         7 Info
PLAT979_ALERT_1_G NoSpherA2 Scattering Factors Used .............     Please Note
```

```
   0 ALERT level A = Most likely a serious problem - resolve or explain
   0 ALERT level B = A potentially serious problem, consider carefully
   2 ALERT level C = Check. Ensure it is not caused by an omission or oversight
  11 ALERT level G = General information/check it is not something unexpected

   3 ALERT type 1 CIF construction/syntax error, inconsistent or missing data
   1 ALERT type 2 Indicator that the structure model may be wrong or deficient
   1 ALERT type 3 Indicator that the structure quality may be low
   6 ALERT type 4 Improvement, methodology, query or suggestion
   2 ALERT type 5 Informative message, check
```

It is advisable to attempt to resolve as many as possible of the alerts in all categories. Often the minor alerts point to easily fixed oversights, errors and omissions in your CIF or refinement strategy, so attention to these fine details can be worthwhile. In order to resolve some of the more serious problems it may be necessary to carry out additional measurements or structure refinements. However, the purpose of your study may justify the reported deviations and the more serious of these should normally be commented upon in the discussion or experimental section of a paper or in the "special_details" fields of the CIF. checkCIF was carefully designed to identify outliers and unusual parameters, but every test has its limitations and alerts that are not important in a particular case may appear. Conversely, the absence of alerts does not guarantee there are no aspects of the results needing attention. It is up to the individual to critically assess their own results and, if necessary, seek expert advice.

**Publication of your CIF in IUCr journals**

A basic structural check has been run on your CIF. These basic checks will be run on all CIFs submitted for publication in IUCr journals (*Acta Crystallographica*, *Journal of Applied Crystallography*, *Journal of Synchrotron Radiation*); however, if you intend to submit to *Acta Crystallographica Section C* or *E* or *IUCrData*, you should make sure that full publication checks are run on the final version of your CIF prior to submission.

**Publication of your CIF in other journals**

Please refer to the *Notes for Authors* of the relevant journal for any special instructions relating to CIF submission.

---

**PLATON version of 11/11/2024; check.def file version of 11/11/2024**

**Datablock YLID_100K_Cu_mono** - ellipsoid plot

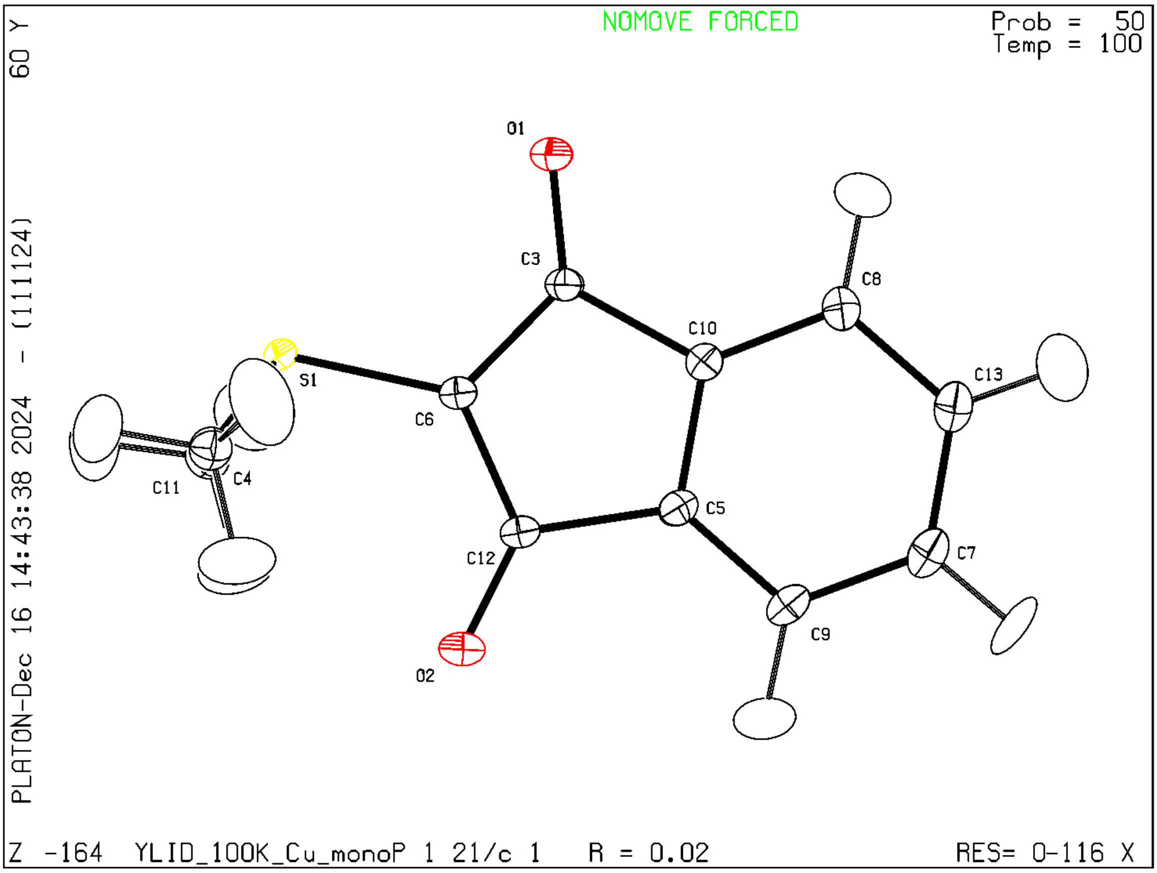

Supplement: Supplementary file 2 — Supplementary Information 2. [file Dataset_25_HAR.pdf › Dataset_25_HAR.pdf]
